# Supplementary material for: Validity and reliability of portable A-mode ultrasound in measuring body fat percentage: A systematic review with meta-analysis
Source: PLoS One. 2024 Feb 8;19(2):e0292872. doi: 10.1371/journal.pone.0292872 (PMC10852247; doi:10.1371/journal.pone.0292872)
Supplement: S1 File — (PDF) [file pone.0292872.s002.pdf]

S1_File. Search phrases

DATABASES	PubMed
**DESCRIPTORS**	Body Composition OR Body Compositions OR Composition, Body OR Compositions, Body AND Ultrasonography OR Echography OR Ultrasound Imaging OR Imaging, Ultrasound OR Imagings, Ultrasound OR Ultrasound Imagings OR Ultrasonic Imaging OR Imaging, Ultrasonic OR Sonography, Medical OR Medical Sonography OR Diagnostic Ultrasound OR Diagnostic Ultrasounds OR Ultrasound, Diagnostic OR Ultrasounds, Diagnostic OR Echotomography OR Diagnosis, Ultrasonic OR Diagnoses, Ultrasonic OR Ultrasonic Diagnoses OR Ultrasonic Diagnosis OR Echotomography, Computer OR Computer Echotomography OR Tomography, Ultrasonic OR Ultrasonic Tomography OR Ultrasonics OR Ultrasonic OR Diagnostic Imaging OR Imaging, Diagnostic OR Medical Imaging OR Imaging, Medical OR "US portable" OR US-portable OR "Ultrasound portable" OR Ultrasound-portable OR Ultrasound waves OR Low intensity pulsed ultrasound* OR Bodymetrix OR Bodymetric*
**PHRASE**	((Body Composition[Title/Abstract] OR Body Compositions[Title/Abstract] OR Composition, Body[Title/Abstract] OR Compositions, Body[Title/Abstract])) AND (Ultrasonography[Title/Abstract] OR Echography[Title/Abstract] OR Ultrasound Imaging[Title/Abstract] OR Imaging, Ultrasound[Title/Abstract] OR Imagings, Ultrasound[Title/Abstract] OR Ultrasound Imagings[Title/Abstract] OR Ultrasonic Imaging[Title/Abstract] OR Imaging, Ultrasonic[Title/Abstract] OR Sonography, Medical[Title/Abstract] OR Medical Sonography[Title/Abstract] OR Diagnostic Ultrasound[Title/Abstract] OR Diagnostic Ultrasounds[Title/Abstract] OR Ultrasound, Diagnostic[Title/Abstract] OR Ultrasounds, Diagnostic[Title/Abstract] OR Echotomography[Title/Abstract] OR Diagnosis, Ultrasonic[Title/Abstract] OR Diagnoses, Ultrasonic[Title/Abstract] OR Ultrasonic Diagnoses[Title/Abstract] OR Ultrasonic Diagnosis[Title/Abstract] OR Echotomography, Computer[Title/Abstract] OR Computer Echotomography[Title/Abstract] OR Tomography, Ultrasonic[Title/Abstract] OR Ultrasonic Tomography[Title/Abstract] OR Ultrasonics[Title/Abstract] OR Ultrasonic[Title/Abstract] OR Diagnostic Imaging[Title/Abstract] OR Imaging, Diagnostic[Title/Abstract] OR Medical Imaging[Title/Abstract] OR Imaging, Medical[Title/Abstract] OR "US portable"[Title/Abstract] OR US-portable[Title/Abstract] OR "Ultrasound portable"[Title/Abstract] OR Ultrasound-portable[Title/Abstract] OR Ultrasound waves[Title/Abstract] OR Low intensity pulsed ultrasound*[Title/Abstract] OR Bodymetrix[Title/Abstract] OR Bodymetric*[Title/Abstract])
**LINK**	https://www.ncbi.nlm.nih.gov/pubmed?term=((Body%20Composition%5BTitle%2FAbstract%5D%20OR%20Body%20Compositions%5BTitle%2FAbstract%5D%20OR%20Composition%2C%20Body%5BTitle%2FAbstract%5D%20OR%20Compositions%2C%20Body%5BTitle%2FAbstract%5D))%20AND%20(Ultrasonography%5BTitle%2FAbstract%5D%20OR%20Echography%5BTitle%2FAbstract%5D%20OR%20Ultrasound%20Imaging%5BTitle%2FAbstract%5D%20OR%20Imaging%2C%20Ultrasound%5BTitle%2FAbstract%5D%20OR%20Imagings%2C%20Ultrasound%5BTitle%2FAbstract%5D%20OR%20Ultrasound%20Imagings%5BTitle%2FAbstract%5D%20OR%20Ultrasonic%20Imaging%5BTitle%2FAbstract%5D%20OR%20Imaging%2C%20Ultrasonic%5BTitle%2FAbstract%5D%20OR%20Sonography%2C%20Medical%5BTitle%2FAbstract%5D%20OR%20Medical%20Sonography%5BTitle%2FAbstract%5D%20OR%20Diagnostic%20Ultrasound%5BTitle%2FAbstract%5D%20OR%20Diagnostic%20Ultrasounds%5BTitle%2FAbstract%5D%20OR%20Ultrasound%2C%20Diagnostic%5BTitle%2FAbstract%5D%20OR%20Ultrasounds%2C%20Diagnostic%5BTitle%2FAbstract%5D%20OR%20Echotomography%5BTitle%2FAbstract%5D%20OR%20Diagnosis%2C%20Ultrasonic%5BTitle%2FAbstract%5D%20OR%20Diagnoses%2C%20Ultrasonic%5BTitle%2FAbstract%5D%20OR%20Ultrasonic%20Diagnoses%5BTitle%2FAbstract%5D%20OR%20Ultrasonic%20Diagnosis%5BTitle%2FAbstract%5D%20OR%20Echotomography%2C%20Computer%5BTitle%2FAbstract%5D%20OR%20Computer%20Echotomography%5BTitle%2FAbstract%5D%20OR%20Tomography%2C%20Ultrasonic%5BTitle%2FAbstract%5D%20OR%20Ultrasonic%20Tomography%5BTitle%2FAbstract%5D%20OR%20Ultrasonics

	%5BTitle%2FAbstract%5D%20OR%20Ultrasonic%5BTitle%2FAbstract%5D%20OR%20Diagnostic%20Imaging%5BTitle%2FAbstract%5D%20OR%20Imaging%2C%20Diagnostic%5BTitle%2FAbstract%5D%20OR%20Medical%20Imaging%5BTitle%2FAbstract%5D%20OR%20Imaging%2C%20Medical%5BTitle%2FAbstract%5D%20OR%20%E2%80%9CUS%20portable%E2%80%9D%5BTitle%2FAbstract%5D%20OR%20US-portable%5BTitle%2FAbstract%5D%20OR%20%E2%80%9CUltrasound%20portable%E2%80%9D%5BTitle%2FAbstract%5D%20OR%20Ultrasound-portable%5BTitle%2FAbstract%5D%20OR%20Ultrasound%20waves%5BTitle%2FAbstract%5D%20OR%20Low%20intensity%20pulsed%20ultrasound*%5BTitle%2FAbstract%5D%20OR%20Bodymetrix%5BTitle%2FAbstract%5D%20OR%20Bodymetric*%5BTitle%2FAbstract%5D)
**SEARCH DATE**	27/05/2022
**SORTED BY**	Best Match
**FILTERS**	Humans
**RESULTS**	619

**DATABASES**	**Cochrane**
**DESCRIPTORS**	Body Composition OR Body Compositions OR Composition, Body OR Compositions, Body AND Ultrasonography OR Echography OR Ultrasound Imaging OR Imaging, Ultrasound OR Imagings, Ultrasound OR Ultrasound Imagings OR Ultrasonic Imaging OR Imaging, Ultrasonic OR Sonography, Medical OR Medical Sonography OR Diagnostic Ultrasound OR Diagnostic Ultrasounds OR Ultrasound, Diagnostic OR Ultrasounds, Diagnostic OR Echotomography OR Diagnosis, Ultrasonic OR Diagnoses, Ultrasonic OR Ultrasonic Diagnoses OR Ultrasonic Diagnosis OR Echotomography, Computer OR Computer Echotomography OR Tomography, Ultrasonic OR Ultrasonic Tomography OR Ultrasonics OR Ultrasonic OR Diagnostic Imaging OR Imaging, Diagnostic OR Medical Imaging OR Imaging, Medical OR "US portable" OR US-portable OR "Ultrasound portable" OR Ultrasound-portable OR Ultrasound waves OR Low intensity pulsed ultrasound* OR Bodymetrix OR Bodymetric*
**PHRASE**	Body Composition OR Body Compositions OR Composition, Body OR Compositions, Body in Title Abstract Keyword AND Ultrasonography OR Echography OR Ultrasound Imaging OR Imaging, Ultrasound OR Imagings, Ultrasound OR Ultrasound Imagings OR Ultrasonic Imaging OR Imaging, Ultrasonic OR Sonography, Medical OR Medical Sonography OR Diagnostic Ultrasound OR Diagnostic Ultrasounds OR Ultrasound, Diagnostic OR Ultrasounds, Diagnostic OR Echotomography OR Diagnosis, Ultrasonic OR Diagnoses, Ultrasonic OR Ultrasonic Diagnoses OR Ultrasonic Diagnosis OR Echotomography, Computer OR Computer Echotomography OR Tomography, Ultrasonic OR Ultrasonic Tomography OR Ultrasonics OR Ultrasonic OR Diagnostic Imaging OR Imaging, Diagnostic OR Medical Imaging OR Imaging, Medical OR "US portable" OR US-portable OR "Ultrasound portable" OR Ultrasound-portable OR Ultrasound waves OR Low intensity pulsed ultrasound* OR Bodymetrix OR Bodymetric* in Title Abstract Keyword *(Word variations have been searched)* Last saved: 12/08/2019 13:15
**LINK**	https://www.cochranelibrary.com/advanced-search?searchType=advanced&database=&status=&publicationYear=&startPublicationYear=&endPublicationYear=&publicationDate=&startPublicationDateYear=&startPublicationDateMonth=&endPublicationDateYear=&endPublicationDateMonth=&wordVariation=&crgs=&controlOptions=AND&searchOptions=1&searchText=&startPublicationDateMonth=1&startPublicationDateYear=&endPublicationDateMonth=1&endPublicationDateYear=&publicationYear=between&startPublicationYear=&endPublicationYear=

**SEARCH DATE**	27/05/2022
**SORTED BY**	Title Abstract Keyword – Cochrane Reviews
**RESULTS**	02

**DATABASES**	**ScienceDirect**
**DESCRIPTORS**	“Body Composition” AND “Validation Studies” AND “Ultrasound Imaging” OR “US portable” OR “US-portable” OR Bodymetrix
**PHRASE**	“Body Composition” AND “Validation Studies” AND “Ultrasound Imaging” OR “US portable” OR “US-portable” OR Bodymetrix
**LINK**	https://www.sciencedirect.com/search/advanced?qs=%E2%80%9CBody%20Composition%E2%80%9D%20AND%20%E2%80%9CValidation%20Studies%E2%80%9D%20AND%20%E2%80%9CUltrasound%20Imaging%E2%80%9D%20OR%20%E2%80%9CUS%20portable%E2%80%9D%20OR%20%E2%80%9CUS-portable%E2%80%9D%20OR%20Bodymetrix&articleTypes=REV%2CFLA&show=25&sortBy=relevance
**SEARCH DATE**	27/05/2022
**SORTED BY**	Title, abstract, keywords
**RESULTS**	43

**DATABASES**	**Scopus**
**DESCRIPTORS**	“Body Composition” AND Ultrasonography OR “Ultrasound Imaging” OR “Diagnostic Ultrasound” OR “US portable” OR “US-portable” OR “Ultrasound portable” OR “Ultrasound-portable” OR “Low intensity pulsed ultrasound*” OR Bodymetrix AND “Validation Studies”
**PHRASE**	“Body Composition” AND Ultrasonography OR “Ultrasound Imaging” OR “Diagnostic Ultrasound” OR “US portable” OR “US-portable” OR “Ultrasound portable” OR “Ultrasound-portable” OR “Low intensity pulsed ultrasound*” OR Bodymetrix AND “Validation Studies”
**LINK**	https://www-scopus.ez422.periodicos.capes.gov.br/results/results.uri?sort=plf-f&src=s&st1=%22Body+Composition%22+AND+Ultrasonography+OR+%22Ultrasound+Imaging%22+OR+%22Diagnostic+Ultrasound%22+OR+%22US+portable%22+OR+%22US-portable%22+OR+%22Ultrasound+portable%22+OR+%22Ultrasound-portable%22+OR+%22Low+intensity+pulsed+ultrasound*%22+OR+Bodymetrix+AND+%22Validation+Studies%22&sid=515a95db025d39c99e4c9ae1ed779192&sot=b&sdt=b&sl=265&s=TITLE-ABS-KEY%28%22Body+Composition%22+AND+Ultrasonography+OR+%22Ultrasound+Imaging%22+OR+%22Diagnostic+Ultrasound%22+OR+%22US+portable%22+OR+%22US-portable%22+OR+%22Ultrasound+portable%22+OR+%22Ultrasound-portable%22+OR+%22Low+intensity+pulsed+ultrasound*%22+OR+Bodymetrix+AND+%22Validation+Studies%22%29&origin=searchbasic&editSaveSearch=&yearFrom=Before+1960&yearTo=Present
**SEARCH DATE**	27/05/2022
**SORTED BY**	TITLE-ABS-KEY
**RESULTS**	16

DATABASES	Web of Science
DESCRIPTORS	(“Body Composition” OR “Body Compositions” OR “Composition, Body” OR “Compositions, Body”) AND ("Ultrasonography" OR "Echography" OR “Ultrasound Imaging” OR “Imaging, Ultrasound” OR “Imagings, Ultrasound” OR “Ultrasound Imagings” OR “Ultrasonic Imaging” OR “Imaging, Ultrasonic” OR “Sonography, Medical” OR “Medical Sonography” OR “Diagnostic Ultrasound” OR “Diagnostic Ultrasounds” OR “Ultrasound, Diagnostic” OR “Ultrasounds, Diagnostic” OR "Echotomography" OR “Diagnosis, Ultrasonic” OR “Diagnoses, Ultrasonic” OR “Ultrasonic Diagnoses” OR “Ultrasonic Diagnosis” OR “Echotomography, Computer” OR “Computer Echotomography” OR “Tomography, Ultrasonic” OR “Ultrasonic Tomography” OR "Ultrasonics" OR "Ultrasonic" OR “Diagnostic Imaging” OR “Imaging, Diagnostic” OR “Medical Imaging” OR “Imaging, Medical” OR “US portable” OR “US-portable” OR “Ultrasound portable” OR “Ultrasound-portable” OR “Ultrasound waves” OR “Low intensity pulsed ultrasound*” OR "bookmetrix" OR "Bodymetric*") AND (“Validation Studies”)
PHRASE	((((("Body Composition" OR "Body Compositions") OR "Composition, Body") OR "Compositions, Body") AND (((((((((((((((((((((((((((((((((((((Ultrasonography OR Echography) OR "Ultrasound Imaging") OR "Imaging, Ultrasound") OR "Imagings, Ultrasound") OR "Ultrasound Imagings") OR "Ultrasonic Imaging") OR "Imaging, Ultrasonic") OR "Sonography, Medical") OR "Medical Sonography") OR "Diagnostic Ultrasound") OR "Diagnostic Ultrasounds") OR "Ultrasound, Diagnostic") OR "Ultrasounds, Diagnostic") OR Echotomography) OR "Diagnosis, Ultrasonic") OR "Diagnoses, Ultrasonic") OR "Ultrasonic Diagnoses") OR "Ultrasonic Diagnosis") OR "Echotomography, Computer") OR "Computer Echotomography") OR "Tomography, Ultrasonic") OR "Ultrasonic Tomography") OR Ultrasonics) OR Ultrasonic) OR "Diagnostic Imaging") OR "Imaging, Diagnostic") OR "Medical Imaging") OR "Imaging, Medical") OR "US portable") OR "US-portable") OR "Ultrasound portable") OR "Ultrasound-portable") OR "Ultrasound waves") OR "Low intensity pulsed ultrasound*") OR bodymetrics) OR Bodymetric*)) AND "Validation Studies")
LINK	https://www.webofscience.com/wos/woscc/summary/dd1833c4-b80f-4ebb-9e13-bc27afad04ab-3ab98701/relevance/1
SEARCH DATE	22/05/2022
SORTED BY	Title
RESULTS	02

DATABASES	LILACS via BVSalud
DESCRIPTORS	body composition AND ultrasound
PHRASE	(tw:((“Body Composition” OR “Body Compositions” OR “Composition, Body” OR “Compositions, Body”) AND (Ultrasonography OR Echography OR “Ultrasound Imaging” OR “Imaging, Ultrasound” OR “Imagings, Ultrasound” OR “Ultrasound Imagings” OR “Ultrasonic Imaging” OR “Imaging, Ultrasonic” OR “Sonography, Medical” OR “Medical Sonography” OR “Diagnostic Ultrasound” OR “Diagnostic Ultrasounds” OR “Ultrasound, Diagnostic” OR “Ultrasounds, Diagnostic” OR Echotomography OR “Diagnosis, Ultrasonic” OR “Diagnoses, Ultrasonic” OR “Ultrasonic Diagnoses” OR “Ultrasonic Diagnosis” OR “Echotomography, Computer” OR “Computer Echotomography” OR “Tomography, Ultrasonic” OR “Ultrasonic Tomography” OR Ultrasonics OR Ultrasonic OR “Diagnostic Imaging” OR “Imaging, Diagnostic” OR “Medical Imaging” OR “Imaging, Medical” OR “US portable” OR “US-portable” OR “Ultrasound portable” OR “Ultrasound-portable” OR “Ultrasound waves” OR “Low intensity pulsed ultrasound*” OR Bodymetrix OR Bodymetric*)))

**LINK**	http://pesquisa.bvsalud.org/portal/?u_filter%5B%5D=fulltext&u_filter%5B%5D=collection&u_filter%5B%5D=db&u_filter%5B%5D=mj_cluster&u_filter%5B%5D=type_of_study&u_filter%5B%5D=clinical_aspect&u_filter%5B%5D=limit&u_filter%5B%5D=pais_assunto&u_filter%5B%5D=la&u_filter%5B%5D=year_cluster&u_filter%5B%5D=type&u_filter%5B%5D=ta_cluster&u_filter%5B%5D=jd&u_filter%5B%5D=pais_afiliacao&fb=&lang=pt&q=%28tw%3A%28%28%E2%80%9CBody+Composition%E2%80%9D+OR+%E2%80%9CBody+Compositions%E2%80%9D+OR+%E2%80%9CComposition%2C+Body%E2%80%9D+OR+%E2%80%9CCompositions%2C+Body%E2%80%9D%29+AND+%28Ultrasonography+OR+Echography+OR+%E2%80%9CUltrasound+Imaging%E2%80%9D+OR+%E2%80%9CImaging%2C+Ultrasound%E2%80%9D+OR+%E2%80%9CImagings%2C+Ultrasound%E2%80%9D+OR+%E2%80%9CUltrasound+Imagings%E2%80%9D+OR+%E2%80%9CUltrasonic+Imaging%E2%80%9D+OR+%E2%80%9CImaging%2C+Ultrasonic%E2%80%9D+OR+%E2%80%9CSonography%2C+Medical%E2%80%9D+OR+%E2%80%9CMedical+Sonography%E2%80%9D+OR+%E2%80%9CDiagnostic+Ultrasound%E2%80%9D+OR+%E2%80%9CDiagnostic+Ultrasounds%E2%80%9D+OR+%E2%80%9CUltrasound%2C+Diagnostic%E2%80%9D+OR+%E2%80%9CUltrasounds%2C+Diagnostic%E2%80%9D+OR+Echotomography+OR+%E2%80%9CDiagnosis%2C+Ultrasonic%E2%80%9D+OR+%E2%80%9CDiagnoses%2C+Ultrasonic%E2%80%9D+OR+%E2%80%9CUltrasonic+Diagnoses%E2%80%9D+OR+%E2%80%9CUltrasonic+Diagnosis%E2%80%9D+OR+%E2%80%9CEchotomography%2C+Computer%E2%80%9D+OR+%E2%80%9CComputer+Echotomography%E2%80%9D+OR+%E2%80%9CTomography%2C+Ultrasonic%E2%80%9D+OR+%E2%80%9CUltrasonic+Tomography%E2%80%9D+OR+Ultrasonics+OR+Ultrasonic+OR+%E2%80%9CDiagnostic+Imaging%E2%80%9D+OR+%E2%80%9CImaging%2C+Diagnostic%E2%80%9D+OR+%E2%80%9CMedical+Imaging%E2%80%9D+OR+%E2%80%9CImaging%2C+Medical%E2%80%9D+OR+%E2%80%9CUS+portable%E2%80%9D+OR+%E2%80%9CUS-portable%E2%80%9D+OR+%E2%80%9CUltrasound+portable%E2%80%9D+OR+%E2%80%9CUltrasound-portable%E2%80%9D+OR+%E2%80%9CUltrasound+waves%E2%80%9D+OR+%E2%80%9CLow+intensity+pulsed+ultrasound*%E2%80%9D+OR+Bodymetrix+OR+Bodymetric*%29%29%29&where=&filter%5Bdb%5D%5B%5D=LILACS
**SEARCH DATE**	22/05/2022
**SORTED BY**	Advanced search Title, abstract, subject and LILACS filter
**RESULTS**	11

**DATABASES**	**SciELO**
**DESCRIPTORS**	body composition AND ultrasound
**PHRASE**	(“body composition” OR “body compositions” OR “composition, body” OR “compositions, body”) AND (ultrasonography OR echography OR “ultrasound imaging” OR “imaging, ultrasound” OR “imagings, ultrasound” OR “ultrasound imagings” OR “ultrasonic imaging” OR “imaging, ultrasonic” OR “sonography, medical” OR “medical sonography” OR “diagnostic ultrasound” OR “diagnostic ultrasounds” OR “ultrasound, diagnostic” OR “ultrasounds, diagnostic” OR echotomography OR “diagnosis, ultrasonic” OR “diagnoses, ultrasonic” OR “ultrasonic diagnoses” OR “ultrasonic diagnosis” OR “echotomography, computer” OR “computer echotomography” OR “tomography, ultrasonic” OR “ultrasonic tomography” OR ultrasonics OR ultrasonic OR “diagnostic imaging” OR “imaging, diagnostic” OR “medical imaging” OR “imaging, medical” OR “us portable” OR “us-portable” OR “ultrasound portable” OR “ultrasound-portable” OR “ultrasound waves” OR “low intensity pulsed ultrasound*” OR bodymetrix OR bodymetric*)
**LINK**	https://search.scielo.org/?q=%28body+composition%29+AND+%28body+composition+AND+ultrasound%29&lang=en&count=15&from=0&output=site&sort=&format=summary&fb=&page=1

**SEARCH DATE**	22/05/2022
**SORTED BY**	Simple search All subjects
**RESULTS**	28

**DATABASES**	**PEDro**
**DESCRIPTORS**	body composition AND ultrasound
**PHRASE**	"body composition" ultrasound
**LINK**	https://search.pedro.org.au/advanced-search/results?abstract_with_title=%22body+composition%22+ultrasound&therapy=0&problem=0&body_part=0&subdiscipline=0&topic=0&method=0&authors_association=&title=&source=&year_of_publication=&date_record_was_created=&nscore=&perpage=20&lop=and&find=&find=Start+Search
**SEARCH DATE**	27/05/2022
**SORTED BY**	Advanced search in title and abstract with boolean AND
**RESULTS**	18

**DATABASES**	**CINAHL**
**DESCRIPTORS**	Body Composition OR Body Compositions OR Composition, Body OR Compositions, Body AND Ultrasonography OR Ultrasound Imaging OR Imaging, Ultrasound OR Imagings, Ultrasound OR Ultrasound Imagings OR Ultrasonic Imaging OR Imaging, Ultrasonic OR Diagnostic Ultrasound OR Diagnostic Ultrasounds OR Ultrasound, Diagnostic OR Ultrasounds, Diagnostic OR Diagnosis, Ultrasonic OR Diagnoses, Ultrasonic OR Ultrasonic Diagnoses OR Ultrasonic Diagnosis OR Ultrasonics OR Ultrasonic OR “US portable” OR US-portable OR “Ultrasound portable” OR Ultrasound-portable OR Ultrasound waves OR Low intensity pulsed ultrasound* OR Bodymetrix OR Bodymetric*
**PHRASE**	SU (Body Composition OR Body Compositions OR Composition, Body OR Compositions, Body) AND (Ultrasonography OR Ultrasound Imaging OR Imaging, Ultrasound OR Imagings, Ultrasound OR Ultrasound Imagings OR Ultrasonic Imaging OR Imaging, Ultrasonic OR Diagnostic Ultrasound OR Diagnostic Ultrasounds OR Ultrasound, Diagnostic OR Ultrasounds, Diagnostic OR Diagnosis, Ultrasonic OR Diagnoses, Ultrasonic OR Ultrasonic Diagnoses OR Ultrasonic Diagnosis OR Ultrasonics OR Ultrasonic OR “US portable” OR US-portable OR “Ultrasound portable” OR Ultrasound-portable OR Ultrasound waves OR Low intensity pulsed ultrasound* OR Bodymetrix OR Bodymetric*)
**LINK**	https://web-s-ebscohost.ez83.periodicos.capes.gov.br/ehost/results?vid=4&sid=f695147a-1df1-4765-984e-9691b00d2583%40redis&bquery=SU+(+Body+Composition+OR+Body+Compositions+OR+Composition%2c+Body+OR+Compositions%2c+Body+)+AND+(+Ultrasonography+OR+Ultrasound+Imaging+OR+Imaging%2c+Ultrasound+OR+Imagings%2c+Ultrasound+OR+Ultrasound+Imagings+OR+Ultrasonic+Imaging+OR+Imaging%2c+Ultrasonic+OR+Diagnostic+Ultrasound+OR+Diagnostic+Ultrasounds+OR+Ultrasound%2c+Diagnostic+OR+Ultrasounds%2c+Diagnostic+OR+Diagnosis%2c+Ultrasonic+OR+Diagnoses%2c+Ultrasonic+OR+Ultrasonic+Diagnoses+OR+Ultrasonic+Diagnosis+OR+Ultrasonics+OR+Ultrasonic+OR+%e2%80%9cUS+portable%e2%80%9d+OR+US-portable+OR+%e2%80%9cUltrasound+portable%e2%80%9d+OR+Ultrasound-portable+OR+Ultrasound+waves+OR+Low+intensity+pulsed+ultrasound*+OR+Body

	metrix+OR+Bodymetric*+)&bdata=JmRiPWM4aCZjbGkwPUZUJmNsdjA9WSZsYW5nPXB0LWJyJnR5cGU9MCZzZWFyY2hNb2RlPVN0YW5kYXJkJnNpdGU9ZWhvc3QtbGl2ZQ%3d%3d
**SEARCH DATE**	31/05/2022
**SORTED BY**	Full text
**RESULTS**	228

**DATABASES**	**SPORTDiscus**
**DESCRIPTORS**	Body Composition OR Body Compositions OR Composition, Body OR Compositions, Body AND Ultrasonography OR Ultrasound Imaging OR Imaging, Ultrasound OR Imagings, Ultrasound OR Ultrasound Imagings OR Ultrasonic Imaging OR Imaging, Ultrasonic OR Diagnostic Ultrasound OR Diagnostic Ultrasounds OR Ultrasound, Diagnostic OR Ultrasounds, Diagnostic OR Diagnosis, Ultrasonic OR Diagnoses, Ultrasonic OR Ultrasonic Diagnoses OR Ultrasonic Diagnosis OR Ultrasonics OR Ultrasonic OR “US portable” OR US-portable OR “Ultrasound portable” OR Ultrasound-portable OR Ultrasound waves OR Low intensity pulsed ultrasound* OR Bodymetrix OR Bodymetric*
**PHRASE**	SU (Body Composition OR Body Compositions OR Composition, Body OR Compositions, Body) AND SU (Ultrasonography OR Ultrasound Imaging OR Imaging, Ultrasound OR Imagings, Ultrasound OR Ultrasound Imagings OR Ultrasonic Imaging OR Imaging, Ultrasonic OR Diagnostic Ultrasound OR Diagnostic Ultrasounds OR Ultrasound, Diagnostic OR Ultrasounds, Diagnostic OR Diagnosis, Ultrasonic OR Diagnoses, Ultrasonic OR Ultrasonic Diagnoses OR Ultrasonic Diagnosis OR Ultrasonics OR Ultrasonic OR “US portable” OR US-portable OR “Ultrasound portable” OR Ultrasound-portable OR Ultrasound waves OR Low intensity pulsed ultrasound* OR Bodymetrix OR Bodymetric*)
**LINK**	https://web-p-ebscohost.ez83.periodicos.capes.gov.br/ehost/results?vid=1&sid=dff95622-1d57-47c1-8d4b-09a2a0c8bfe1%40redis&bquery=SU+(+Body+Composition+OR+Body+Compositions+OR+Composition%2c+Body+OR+Compositions%2c+Body+)+AND+SU+(+Ultrasonography+OR+Ultrasound+Imaging+OR+Imaging%2c+Ultrasound+OR+Imagings%2c+Ultrasound+OR+Ultrasound+Imagings+OR+Ultrasonic+Imaging+OR+Imaging%2c+Ultrasonic+OR+Diagnostic+Ultrasound+OR+Diagnostic+Ultrasounds+OR+Ultrasound%2c+Diagnostic+OR+Ultrasounds%2c+Diagnostic+OR+Diagnosis%2c+Ultrasonic+OR+Diagnoses%2c+Ultrasonic+OR+Ultrasonic+Diagnoses+OR+Ultrasonic+Diagnosis+OR+Ultrasonics+OR+Ultrasonic+OR+%e2%80%9cUS+portable%e2%80%9d+OR+US-portable+OR+%e2%80%9cUltrasound+portable%e2%80%9d+OR+Ultrasound-portable+OR+Ultrasound+waves+OR+Low+intensity+pulsed+ultrasound*+OR+Bodymetrix+OR+Bodymetric*+)&bdata=JmRiPXMzaCZjbGkwPUZUJmNsdjA9WSZsYW5nPXB0LWJyJnR5cGU9MCZzZWFyY2hNb2RlPVN0YW5kYXJkJnNpdGU9ZWhvc3QtbGl2ZQ%3d%3d
**SEARCH DATE**	31/05/2022
**SORTED BY**	Full text
**RESULTS**	28

RESULTS

DATABASES	SEARCH DATE	RESULTS
**PubMed**	27/05/2022	619
**Cochrane**	27/05/2022	2
**ScienceDirect**	27/05/2022	43
**Scopus**	27/05/2022	16
**Web of Science**	22/05/2022	2
**LILACS via BVSalud**	22/05/2022	11
**SciELO**	22/05/2022	28
**PEDro**	27/05/2022	18
**CINAHL**	31/05/2022	228
**SPORTDiscus**	31/05/2022	28
**TOTAL**		995
