# Supplementary material for: Validity and reliability of portable A-mode ultrasound in measuring body fat percentage: A systematic review with meta-analysis
Source: PLoS One. 2024 Feb 8;19(2):e0292872. doi: 10.1371/journal.pone.0292872 (PMC10852247; doi:10.1371/journal.pone.0292872)
Supplement: S2 File — (PDF) [file pone.0292872.s003.pdf]

# QAREL

| | |
|---|---|
| **TITLE** | Estimation of body fat in adults using a portable A-mode ultrasound |
| **AUTHOR** | Renata M. Bielemann Ph.D. a,b , Maria Cristina Gonzalez Ph.D. c,*, Thiago Gonzalez Barbosa-Silva M.Sc . a, Silvana Paiva Orlandi Ph.D. b, Mariana Otero Xavier CN a, Rafaela Bülow Bergmann M.Sc. _ c, Maria Cec ılia Formoso Assunc ¸ ~ao Ph.D. |
| **OBJECTIVE** | This study aimed to develop and validate equations to estimate body fat based on anthropometric measurements of subcutaneous fat thickness (SFT) and muscle thickness (MT) measured by A-mode ultrasound ( BodyMetrix ) in Brazilian adults. |

| | **Item** | **Yes** | **No** | **Unclear** | **N/A** | **Excluded** |
|---|---|---|---|---|---|---|
| 1. | Was the test evaluated in a sample of subjects who were representative of those to whom theauthors intended the results to be applied? (DEF: 3, 4, 5, 7, 8, 9) | | X | | | |
| 2. | Was the test performed by raters who were representative of those to whom the authorsintended the results to be applied? (DEF 3, 4, 6, 7, 8, 9) | | | X | | |
| 3. | Were raters blinded to the findings of other raters during the study?(DEF 10) | | | | | X |
| 4. | Were raters blinded to their own prior findings of the test under evaluation?(DEF 11) | | | | | X |
| 5. | Were raters blinded to the results of the reference standard for the target disorder(or variable) being evaluated? (DEF 12) | | | | | X |
| 6. | Were raters blinded to clinical information that was not intended to be provided as part of thetesting procedure or study design? (DEF 13) | | | | | X |
| 7. | Were raters blinded to additional cues that were not part of the test?(DEF 14) | | | | | X |
| 8. | Was the order of examination varied?(DEF 15, 16) | | | | | X |
| 9. | Was the time interval between repeated measurements compatible with the stability (ortheoretical stability) of the variable being measured? (DEF 17) | | | X | | |
| 10. | Was the test applied correctly and interpreted appropriately?(DEF 18) | X | | | | |
| 11. | Were appropriate statistical measures of agreement used?(DEF 19, 20, 21) | X | | | | |

# QAREL

| | |
|---|---|
| **TITLE** | Reliability and Validity of A-Mode Ultrasound to Quantify Body Composition |
| **AUTHOR** | Nathan Hendrickson, MD, John Davison, MPH, Luke Schiller, BS, and Michael Willey, MD |
| **OBJECTIVE** | The purpose of this study was to assess reliability and validity of amplitude-mode (A-mode) ultrasound (US) to quantify body composition for use in a randomized clinical trial assessing interventions to prevent muscle loss in young individuals after high-energy musculoskeletal trauma. |

| Item | | Yes | No | Unclear | N/A | Excluded |
|---|---|---|---|---|---|---|
| 1. | Was the test evaluated in a sample of subjects who were representative of those to whom theauthors intended the results to be applied? (DEF: 3, 4, 5, 7, 8, 9) | | X | | | |
| 2. | Was the test performed by raters who were representative of those to whom the authorsintended the results to be applied? (DEF 3, 4, 6, 7, 8, 9) | X | | | | |
| 3. | Were raters blinded to the findings of other raters during the study?(DEF 10) | | | | | X |
| 4. | Were raters blinded to their own prior findings of the test under evaluation?(DEF 11) | | | | | X |
| 5. | Were raters blinded to the results of the reference standard for the target disorder(or variable) being evaluated? (DEF 12) | | | | | X |
| 6. | Were raters blinded to clinical information that was not intended to be provided as part of thetesting procedure or study design? (DEF 13) | | | | | X |
| 7. | Were raters blinded to additional cues that were not part of the test?(DEF 14) | | | | | X |
| 8. | Was the order of examination varied?(DEF 15, 16) | | | | | X |
| 9. | Was the time interval between repeated measurements compatible with the stability (ortheoretical stability) of the variable being measured? (DEF 17) | X | | | | |
| 10. | Was the test applied correctly and interpreted appropriately?(DEF 18) | | X | | | |
| 11. | Were appropriate statistical measures of agreement used?(DEF 19, 20, 21) | X | | | | |

## QAREL

| | |
|---|---|
| **TITLE** | Agreement between ultrasound and dual-energy X-ray absorptiometry in assessing percentage body fat in college-aged adults |
| **AUTHOR** | Kelly E. Johnson1,2, Brian Miller1, Judith A. Juvancic-Heltzel1, Sarah E. Agnor1, Dana L. Kiger1, Rachele M. Kappler1 and Ronald Otterstetter |
| **OBJECTIVE** | To employ a cross-over design to investigate the agreement between ULTRA using a seven-site methodology and DXA in assessing body fat (%BF) in a normal-weight sample of college-age men and women |

| **Item** | | **Yes** | **No** | **Unclear** | **N/A** | **Excluded** |
|---|---|---|---|---|---|---|
| 1. | Was the test evaluated in a sample of subjects who were representative of those to whom theauthors intended the results to be applied? (DEF: 3, 4, 5, 7, 8, 9) | | X | | | |
| 2. | Was the test performed by raters who were representative of those to whom the authorsintended the results to be applied? (DEF 3, 4, 6, 7, 8, 9) | | | X | | |
| 3. | Were raters blinded to the findings of other raters during the study?(DEF 10) | | | | | X |
| 4. | Were raters blinded to their own prior findings of the test under evaluation?(DEF 11) | | | | | X |
| 5. | Were raters blinded to the results of the reference standard for the target disorder(or variable) being evaluated? (DEF 12) | | | | | X |
| 6. | Were raters blinded to clinical information that was not intended to be provided as part of thetesting procedure or study design? (DEF 13) | | | | | X |
| 7. | Were raters blinded to additional cues that were not part of the test?(DEF 14) | | | | | X |
| 8. | Was the order of examination varied?(DEF 15, 16) | | | | | X |
| 9. | Was the time interval between repeated measurements compatible with the stability (ortheoretical stability) of the variable being measured? (DEF 17) | X | | | | |
| 10. | Was the test applied correctly and interpreted appropriately?(DEF 18) | X | | | | |
| 11. | Were appropriate statistical measures of agreement used?(DEF 19, 20, 21) | X | | | | |

# QAREL

| | |
|---|---|
| **TITLE** | Validity of the Portable Ultrasound BodyMetrix — BX-2000 for Measuring Body Fat Percentage<br><br>Validity of BodyMetrix Portable Ultrasound BX-2000 for Body Fat Percentage Measurement |
| **AUTHOR** | KANG ET AL., 2020 |
| **OBJECTIVE** | Systemically investigate possible errors in different protocols and demonstrate the validity of %BF between each different protocol in BodyMetrix compared to the criterion method (ie DEXA) through further statistical analysis (ie Equivalence Test). |

| Item | | Yes | No | Unclear | N/A | Excluded |
|---|---|---|---|---|---|---|
| 1. | Was the test evaluated in a sample of subjects who were representative of those to whom theauthors intended the results to be applied? (DEF: 3, 4, 5, 7, 8, 9) | | X | | | |
| 2. | Was the test performed by raters who were representative of those to whom the authorsintended the results to be applied? (DEF 3, 4, 6, 7, 8, 9) | | | X | | |
| 3. | Were raters blinded to the findings of other raters during the study?(DEF 10) | | | | | X |
| 4. | Were raters blinded to their own prior findings of the test under evaluation?(DEF 11) | | | | | X |
| 5. | Were raters blinded to the results of the reference standard for the target disorder(or variable) being evaluated? (DEF 12) | | | | | X |
| 6. | Were raters blinded to clinical information that was not intended to be provided as part of thetesting procedure or study design? (DEF 13) | | | | | X |
| 7. | Were raters blinded to additional cues that were not part of the test?(DEF 14) | | | | | X |
| 8. | Was the order of examination varied?(DEF 15, 16) | | | | | X |
| 9. | Was the time interval between repeated measurements compatible with the stability (ortheoretical stability) of the variable being measured? (DEF 17) | | | X | | |
| 10. | Was the test applied correctly and interpreted appropriately?(DEF 18) | X | | | | |
| 11. | Were appropriate statistical measures of agreement used?(DEF 19, 20, 21) | X | | | | |

# QAREL

| | |
|---|---|
| **TITLE** | Fat Percentage Evaluation Through Portable Ultrasound in Adolescents: A Comparison with Dual-energy X-ray Absorptiometry |
| **AUTHOR** | wagner luis Ripka , Pedro Miguel Gewehr , Leandra Ulbricht |
| **OBJECTIVE** | Analyzing the performance of portable US, in comparison with dual-energy X-ray absorptiometry (DXA) reference method, in specific equations for predicting fat percentage in male adolescents |

| Item | | Yes | No | Unclear | N/A | Excluded |
|---|---|---|---|---|---|---|
| 1. | Was the test evaluated in a sample of subjects who were representative of those to whom theauthors intended the results to be applied? (DEF: 3, 4, 5, 7, 8, 9) | | X | | | |
| 2. | Was the test performed by raters who were representative of those to whom the authorsintended the results to be applied? (DEF 3, 4, 6, 7, 8, 9) | | | X | | |
| 3. | Were raters blinded to the findings of other raters during the study?(DEF 10) | | | | | X |
| 4. | Were raters blinded to their own prior findings of the test under evaluation?(DEF 11) | | | | | X |
| 5. | Were raters blinded to the results of the reference standard for the target disorder(or variable) being evaluated? (DEF 12) | | | | | X |
| 6. | Were raters blinded to clinical information that was not intended to be provided as part of thetesting procedure or study design? (DEF 13) | | | | | X |
| 7. | Were raters blinded to additional cues that were not part of the test?(DEF 14) | | | | | X |
| 8. | Was the order of examination varied?(DEF 15, 16) | | | | | X |
| 9. | Was the time interval between repeated measurements compatible with the stability (ortheoretical stability) of the variable being measured? (DEF 17) | | | X | | |
| 10. | Was the test applied correctly and interpreted appropriately?(DEF 18) | | X | | | |
| 11. | Were appropriate statistical measures of agreement used?(DEF 19, 20, 21) | X | | | | |

# QAREL

| | |
|---|---|
| **TITLE** | Portable A-Mode Ultrasound for Body Composition Assessment in Adolescents |
| **AUTHOR** | wagner luis Ripka , Leandra Ulbricht , Lucas Menghin , Pedro Miguel Gewehr |
| **OBJECTIVE** | Assess the agreement of portable US with a reference standard method, dual-energy x-ray absorptiometry (DXA), for body fat percentage (BF%) in adolescents and verify whether the use of a new mathematical model, based on the anatomical thickness obtained by the US, is capable of improving BF% prediction. |

| Item | | Yes | No | Uncl ear | N/A | Excl uded |
|---|---|---|---|---|---|---|
| 1. | Was the test evaluated in a sample of subjects who were representative of those to whom theauthors intended the results to be applied? (DEF: 3, 4, 5, 7, 8, 9) | | X | | | |
| 2. | Was the test performed by raters who were representative of those to whom the authorsintended the results to be applied? (DEF 3, 4, 6, 7, 8, 9) | | | X | | |
| 3. | Were raters blinded to the findings of other raters during the study?(DEF 10) | | | | | X |
| 4. | Were raters blinded to their own prior findings of the test under evaluation?(DEF 11) | | | | | X |
| 5. | Were raters blinded to the results of the reference standard for the target disorder(or variable) being evaluated? (DEF 12) | | | | | X |
| 6. | Were raters blinded to clinical information that was not intended to be provided as part of thetesting procedure or study design? (DEF 13) | | | | | X |
| 7. | Were raters blinded to additional cues that were not part of the test?(DEF 14) | | | | | X |
| 8. | Was the order of examination varied?(DEF 15, 16) | | | | | X |
| 9. | Was the time interval between repeated measurements compatible with the stability (ortheoretical stability) of the variable being measured? (DEF 17) | | | X | | |
| 10. | Was the test applied correctly and interpreted appropriately?(DEF 18) | X | | | | |
| 11. | Were appropriate statistical measures of agreement used?(DEF 19, 20, 21) | X | | | | |

**QAREL**

| | |
|---|---|
| **TITLE** | Comparison of amplitude-mode ultrasound versus air displacement plethysmography for assessing body composition changes following participation in a structured weight-loss program in women |
| **AUTHOR** | Brad J. Schoenfeld1, Alan A. Aragon2, Jordan Moon3, James W. Krieger4 and Gul Tiryaki-Sonmez1 |
| **OBJECTIVE** | Compare body composition changes as measured by A-mode ultrasound (US) versus a criterion densitometry-based measure, air displacement plethysmography (ADP) |

| **Item** | **Yes** | **No** | **Unclear** | **N/A** | **Excluded** |
|---|---|---|---|---|---|
| 1. Was the test evaluated in a sample of subjects who were representative of those to whom theauthors intended the results to be applied? (DEF: 3, 4, 5, 7, 8, 9) | | X | | | |
| 2. Was the test performed by raters who were representative of those to whom the authorsintended the results to be applied? (DEF 3, 4, 6, 7, 8, 9) | X | | | | |
| 3. Were raters blinded to the findings of other raters during the study?(DEF 10) | | | | | X |
| 4. Were raters blinded to their own prior findings of the test under evaluation?(DEF 11) | | | | | X |
| 5. Were raters blinded to the results of the reference standard for the target disorder(or variable) being evaluated? (DEF 12) | | | | | X |
| 6. Were raters blinded to clinical information that was not intended to be provided as part of thetesting procedure or study design? (DEF 13) | | | | | X |
| 7. Were raters blinded to additional cues that were not part of the test?(DEF 14) | | | | | X |
| 8. Was the order of examination varied?(DEF 15, 16) | | | | | X |
| 9. Was the time interval between repeated measurements compatible with the stability (ortheoretical stability) of the variable being measured? (DEF 17) | | | X | | |
| 10. Was the test applied correctly and interpreted appropriately?(DEF 18) | | X | | | |
| 11. Were appropriate statistical measures of agreement used?(DEF 19, 20, 21) | X | | | | |

# QAREL

| | |
|---|---|
| **TITLE** | Reproducibility and Validity of A-Mode Ultrasound for Body Composition Measurement and Classification in Overweight and Obese Men and Women |
| **AUTHOR** | Abbie E. Smith-Ryan*, Sarah N. Fultz, Malia N. Melvin, Hailee L. Wingfield, Mary N. Woessner |
| **OBJECTIVE** | To compare the validity and reliability of a portable A-mode ultrasound (US) to a criterion three compartment model (3C) for the measurement of body composition. |

| Item | | Yes | No | Unclear | N/A | Excluded |
|---|---|---|---|---|---|---|
| 1. | Was the test evaluated in a sample of subjects who were representative of those to whom theauthors intended the results to be applied? (DEF: 3, 4, 5, 7, 8, 9) | | X | | | |
| 2. | Was the test performed by raters who were representative of those to whom the authorsintended the results to be applied? (DEF 3, 4, 6, 7, 8, 9) | X | | | | |
| 3. | Were raters blinded to the findings of other raters during the study?(DEF 10) | | | | | X |
| 4. | Were raters blinded to their own prior findings of the test under evaluation?(DEF 11) | | | | | X |
| 5. | Were raters blinded to the results of the reference standard for the target disorder(or variable) being evaluated? (DEF 12) | | | | | X |
| 6. | Were raters blinded to clinical information that was not intended to be provided as part of thetesting procedure or study design? (DEF 13) | | | | | X |
| 7. | Were raters blinded to additional cues that were not part of the test?(DEF 14) | | | | | X |
| 8. | Was the order of examination varied?(DEF 15, 16) | | | | | X |
| 9. | Was the time interval between repeated measurements compatible with the stability (ortheoretical stability) of the variable being measured? (DEF 17) | X | | | | |
| 10. | Was the test applied correctly and interpreted appropriately?(DEF 18) | | X | | | |
| 11. | Were appropriate statistical measures of agreement used?(DEF 19, 20, 21) | X | | | | |

# QAREL

| | |
|---|---|
| **TITLE** | Test-retest reliability and validity of body composition methods in adults<br><br>retest reliability and validity of body composition methods in adults |
| **AUTHOR** | TOTOSY DE ZEPETNEK et al., 2021 |
| **OBJECTIVE** | to evaluate the test-retest reliability and validity of three body composition measurement devices. |

| **Item** | | **Yes** | **No** | **Unclear** | **N/A** | **Excluded** |
|---|---|---|---|---|---|---|
| 1. | Was the test evaluated in a sample of subjects who were representative of those to whom theauthors intended the results to be applied? (DEF: 3, 4, 5, 7, 8, 9) | | **X** | | | |
| 2. | Was the test performed by raters who were representative of those to whom the authorsintended the results to be applied? (DEF 3, 4, 6, 7, 8, 9) | | | **X** | | |
| 3. | Were raters blinded to the findings of other raters during the study?(DEF 10) | | | | | **X** |
| 4. | Were raters blinded to their own prior findings of the test under evaluation?(DEF 11) | | | | | **X** |
| 5. | Were raters blinded to the results of the reference standard for the target disorder(or variable) being evaluated? (DEF 12) | | | | | **X** |
| 6. | Were raters blinded to clinical information that was not intended to be provided as part of thetesting procedure or study design? (DEF 13) | | | | | **X** |
| 7. | Were raters blinded to additional cues that were not part of the test?(DEF 14) | | | | | **X** |
| 8. | Was the order of examination varied?(DEF 15, 16) | | | | | **X** |
| 9. | Was the time interval between repeated measurements compatible with the stability (ortheoretical stability) of the variable being measured? (DEF 17) | **X** | | | | |
| 10. | Was the test applied correctly and interpreted appropriately?(DEF 18) | | **X** | | | |
| 11. | Were appropriate statistical measures of agreement used?(DEF 19, 20, 21) | **X** | | | | |

# QAREL

| | |
|---|---|
| **TITLE** | Validity and Reliability of A-Mode Ultrasound for Body Composition Assessment of NCAA Division I Athletes |
| **AUTHOR** | Dale R. Wagner*, Dustin L. Cain, Nicolas W. Clark |
| **OBJECTIVE** | This study evaluated the validity and reliability of the BodyMetrix™BX2000 A-mode ultrasound for estimating percent body fat (%BF) in athletes by comparing it to skinfolds and the BOD POD |

| Item | | Yes | No | Unclear | N/A | Excluded |
|---|---|---|---|---|---|---|
| 1. | Was the test evaluated in a sample of subjects who were representative of those to whom theauthors intended the results to be applied? (DEF: 3, 4, 5, 7, 8, 9) | | X | | | |
| 2. | Was the test performed by raters who were representative of those to whom the authorsintended the results to be applied? (DEF 3, 4, 6, 7, 8, 9) | X | | | | |
| 3. | Were raters blinded to the findings of other raters during the study?(DEF 10) | | | | | X |
| 4. | Were raters blinded to their own prior findings of the test under evaluation?(DEF 11) | | | | | X |
| 5. | Were raters blinded to the results of the reference standard for the target disorder(or variable) being evaluated? (DEF 12) | | | | | X |
| 6. | Were raters blinded to clinical information that was not intended to be provided as part of thetesting procedure or study design? (DEF 13) | | | | | X |
| 7. | Were raters blinded to additional cues that were not part of the test?(DEF 14) | | | | | X |
| 8. | Was the order of examination varied?(DEF 15, 16) | | | | | X |
| 9. | Was the time interval between repeated measurements compatible with the stability (ortheoretical stability) of the variable being measured? (DEF 17) | X | | | | |
| 10. | Was the test applied correctly and interpreted appropriately?(DEF 18) | | X | | | |
| 11. | Were appropriate statistical measures of agreement used?(DEF 19, 20, 21) | X | | | | |
