# Supplementary material for: Validity and reliability of portable A-mode ultrasound in measuring body fat percentage: A systematic review with meta-analysis
Source: PLoS One. 2024 Feb 8;19(2):e0292872. doi: 10.1371/journal.pone.0292872 (PMC10852247; doi:10.1371/journal.pone.0292872)
Supplement: S3 File — (PDF) [file pone.0292872.s004.pdf]

# QUADAS-2

| | |
|---|---|
| **TITLE** | Seven-site versus three-site method of body composition using BodyMetrix ultrasound compared to dual-energy X-ray absorptiometry |
| **AUTHOR** | Marissa N. Baranauskas1, Kelly E. Johnson1,2, Judith A. Juvancic-Heltzel1, Rachele M. Kappler1, Laura Richardson1, Scott Jamieson1 and Ronald Otterstetter |
| **OBJECTIVE** | The purpose of the study was twofold ( i ) to compare agreement between Jackson–Pollock seven site (JP7), Jackson–Pollock three site (JP3), and Pollock three site (P3) to predict body fat (%BF) using ULTRA and ( ii) and to compare the three ULTRA methods against DXA |

## PHASE 1: Formulate the review research question

***Note:*** *You should report your systematic review question in terms of patients, index test(s) and reference standard, and target condition. As the accuracy of a test can depend on where the diagnostic pathway will be used; describe the patients in terms of setup, intended use of the index test, patient presentation, and previous tests.*

| **Patients: (clinical setting, intended use of index test, presentation, previous testing)** |
|---|
| N=76 university students, 33 men, and 43 women<br>Measured by BM and then by DEXA and performed on the same day<br>Average BMI of 24 |
| **Index test(s)** |
| BM |
| **Reference Standard and target condition** |
| DEXA |

## Phase 2: Draw the flowchart for the primary study

***Note:*** *It is to evaluate the published flow diagram for the primary study or draw one if none is reported or if the published diagram is not adequate. The flowchart will facilitate judgment of risk of bias and should provide information on the method of patient recruitment (e.g. based on a consecutive series of patients with specific symptoms suspected of having the target condition, or of cases and controls ), the test execution order and the number of patients submitted to the index test and the reference standard. A hand-drawn diagram is sufficient as this step need not be reported as part of the QUADAS-2 assessment.*

| |
|---|
| |

## Phase 3: Risk of bias and applicability judgment

## DOMAIN 1: PATIENT SELECTION

| Describe the patients included (prior testing, presentation, the intended use of the index test, and clinical setting | | | |
|---|---|---|---|
| **signaling issues** | **YES** | **NO** | **UNCERTAIN** |
| Were patients recruited consecutively or through random sampling? | | X | |
| Was case-control design avoided? | X | | |
| Did the study avoid inappropriate exclusions? | X | | |
| **1) Risk of bias** | **LOW** | **HIGH** | **UNCERTAIN** |
| Could patient selection have introduced biases? | | X | |

| COLLEGE STUDENTS WITH AVERAGE BMI OF 24 | | | |
|---|---|---|---|
| **2) Concerns related to applicability** | **LOW** | **HIGH** | **UNCERTAIN** |
| Is there a concern that the patients included do not match the research question?<br>COLLEGE STUDENTS WITH AVERAGE BMI OF 24 | | x | |

## DOMAIN 2: INDEX TEST(S) (if more than one index test is used, one must be completed for each test)

| Describe the patients included (prior testing, presentation, the intended use of the index test, and clinical setting | | | |
|---|---|---|---|
| **signaling issues** | **YES** | **NO** | **UNCERTAIN** |
| Were the index test results interpreted without knowledge of the reference standard results? | X | | |
| Was a positivity threshold used? Was it pre-specified? | | X | |
| **1) Risk of bias** | **LOW** | **HIGH** | **UNCERTAIN** |
| Could the conduct or interpretation of the index test have introduced biases? | X | | |
| **2) Concerns related to applicability** | **LOW** | **HIGH** | **UNCERTAIN** |
| Is there a concern that the index test, its conduct, or interpretation differs from the review research question?<br>BM EVALUATES THE %F | X | | |

## DOMAIN 3: REFERENCE STANDARD

| Describe the patients included (prior testing, presentation, the intended use of the index test, and clinical setting | | | |
|---|---|---|---|
| **signaling issues** | **YES** | **NO** | **UNCERTAIN** |
| Did the reference standard likely correctly classify the target condition? | X | | |
| Were the reference standard results interpreted without knowledge of the index test results? | X | | |
| **1) Risk of bias** | **LOW** | **HIGH** | **UNCERTAIN** |
| Could the reference standard, its conduct, or interpretation of the index test have introduced biases? | X | | |
| **2) Concerns related to applicability** | **LOW** | **HIGH** | **UNCERTAIN** |
| Is there a concern that the target condition defined by the reference standard does not match the research question?<br>EVALUATING THE %G | X | | |

## DOMAIN 4: TIME FLOW

| Please describe any patient who did not receive the index test(s) and/or the reference standard or who were excluded from the 2 x 2 table (refer to the Flowchart) | | | |
|---|---|---|---|
| **signaling issues** | **YES** | **NO** | **UNCERTAIN** |
| 1. There was an appropriate time interval between the application of the index test(s) and the reference standard<br>ON THE SAME DAY | | | x |
| 2. Did all patients receive the reference standard? | X | | |
| 3. Did the patients receive the same reference standard? | X | | |
| 4. Were all patients included in the analyses? | X | | |
| **1) Risk of bias** | **LOW** | **HIGH** | **UNCERTAIN** |
| Could patient flow have introduced bias? | | | x |

# QUADAS-2

| | |
|---|---|
| **TITLE** | Estimation of body fat in adults using a portable A-mode ultrasound |
| **AUTHOR** | Renata M. Bielemann Ph.D. a,b , Maria Cristina Gonzalez Ph.D. c,*, Thiago Gonzalez Barbosa-Silva M.Sc . a, Silvana Paiva Orlandi Ph.D. b, Mariana Otero Xavier CN a, Rafaela Bülow Bergmann M.Sc. _ c, Maria Cec ılia Formoso Assunc ¸ ~ao Ph.D. |
| **OBJECTIVE** | This study aimed to develop and validate equations to estimate body fat based on anthropometric measurements of subcutaneous fat thickness (SFT) and muscle thickness (MT) measured by A-mode ultrasound ( BodyMetrix ) in Brazilian adults. |

## PHASE 1: Formulate the review research question

***Note:*** *You should report your systematic review question in terms of patients, index test(s) and reference standard, and target condition. As the accuracy of a test can depend on where the diagnostic pathway will be used; describe the patients in terms of setup, intended use of the index test, patient presentation, and previous tests.*

| **Patients: (clinical setting, intended use of index test, presentation, previous testing)** |
|---|
| N=206 being 104 women. 49 women and 51 men had a BMI over 25<br>Measured by Plethysmograph and then by BM |
| **Index test(s)** |
| BM |
| **Reference Standard and target condition** |
| plethysmograph |

## Phase 2: Draw the flowchart for the primary study

***Note:*** *It is to evaluate the published flow diagram for the primary study or draw one if none is reported or if the published diagram is not adequate. The flowchart will facilitate judgment of risk of bias and should provide information on the method of patient recruitment (e.g. based on a consecutive series of patients with specific symptoms suspected of having the target condition, or of cases and controls ), the test execution order and the number of patients submitted to the index test and the reference standard. A hand-drawn diagram is sufficient as this step need not be reported as part of the QUADAS-2 assessment.*

| |
|---|
| |

## Phase 3: Risk of bias and applicability judgment

## DOMAIN 1: PATIENT SELECTION

| Describe the patients included (prior testing, presentation, intended use of the index test, and clinical setting | | | |
|---|---|---|---|
| **signaling issues** | **YES** | **NO** | **UNCERTAIN** |
| Were patients recruited consecutively or through random sampling? | | X | |
| Was case-control design avoided? | X | | |
| Did the study avoid inappropriate exclusions? | X | | |
| **1) Risk of bias** | **LOW** | **HIGH** | **UNCERTAIN** |
| Could patient selection have introduced biases?<br>MEN AND WOMEN WITH AN AVERAGE BMI OF 25 | | X | |

| 2) Concerns related to applicability | LOW | HIGH | UNCERTAIN |
|---|---|---|---|
| Is there a concern that the patients included do not match the research question?<br>MEN AND WOMEN WITH AN AVERAGE BMI OF 25 | X | | |

**DOMAIN 2: INDEX TEST(S)** (if more than one index test is used, one must be completed for each test)

| Describe the patients included (prior testing, presentation, intended use of the index test, and clinical setting | | | |
|---|---|---|---|
| **signaling issues** | **YES** | **NO** | **UNCERTAIN** |
| Were the index test results interpreted without knowledge of the reference standard results? | | X | |
| Was a positivity threshold used? Was it pre-specified? | | X | |
| **1) Risk of bias** | **LOW** | **HIGH** | **UNCERTAIN** |
| Could the conduct or interpretation of the index test have introduced biases? | | X | |
| **2) Concerns related to applicability** | **LOW** | **HIGH** | **UNCERTAIN** |
| Is there a concern that the index test, its conduct or interpretation differs from the review research question?<br>BM EVALUATES THE %F | X | | |

**DOMAIN 3: REFERENCE STANDARD**

| Describe the patients included (prior testing, presentation, intended use of the index test, and clinical setting | | | |
|---|---|---|---|
| **signaling issues** | **YES** | **NO** | **UNCERTAIN** |
| Did the reference standard likely correctly classify the target condition? | X | | |
| Were the reference standard results interpreted without knowledge of the index test results? | X | | |
| **1) Risk of bias** | **LOW** | **HIGH** | **UNCERTAIN** |
| Could the reference standard, its conduct or interpretation of the index test have introduced biases? | X | | |
| **2) Concerns related to applicability** | **LOW** | **HIGH** | **UNCERTAIN** |
| Is there a concern that the target condition defined by the reference standard does not match the research question?<br>EVALUATING THE %G | X | | |

**DOMAIN 4: TIME FLOW**

| Please describe any patient who did not receive the index test(s) and/or the reference standard or who were excluded from the 2 x 2 table (refer to the Flowchart)<br>Describe the time lag and any intervention between the index test(s) and the reference standard | | | |
|---|---|---|---|
| **signaling issues** | **YES** | **NO** | **UNCERTAIN** |
| 1. There was an appropriate time interval between the application of the index test(s) and the reference standard | | | X |
| 2. Did all patients receive the reference standard? | X | | |
| 3. Did the patients receive the same reference standard? | X | | |
| 4. Were all patients included in the analyses? | X | | |
| **1) Risk of bias** | **LOW** | **HIGH** | **UNCERTAIN** |
| Could patient flow have introduced bias? | | | X |

# QUADAS-2

| | |
|---|---|
| **TITLE** | Validation of Three Body Composition Techniques with a Comparison of Ultrasound Abdominal Fat Depths against an Octopolar Bioelectrical Impedance Device |
| **AUTHOR** | KELLY E. JOHNSON*, IAN A. NACCARATO*, MICHELLE A. CORDER*, and WENDY ES REPOVICH |
| **OBJECTIVE** | Were to cross-validate three clinical-grade measures of body composition, using an octopolar Bioelectrical Impedance (BIA), an ultrasound analyzer (US), and Air-Displacement Plethysmography (ADP) and second to compare the US scans of total abdominal, subcutaneous and visceral fat depths (mm) against the trunk percent fat (%BF) from the octopolar BIA |

## PHASE 1: Formulate the review research question

***Note:*** *You should report your systematic review question in terms of patients, index test(s) and reference standard, and target condition. As the accuracy of a test can depend on where the diagnostic pathway will be used; describe the patients in terms of setup, intended use of the index test, patient presentation, and previous tests.*

| **Patients: (clinical setting, intended use of index test, presentation, previous testing)** |
|---|
| N=26 university students, 18 men, and 8 women.<br>The 3 measurements in this sequence ( Plethysmograph first, BIO, and BM randomly allocated)<br>All were evaluated by the index test and the reference standard |
| **Index test(s)** |
| BM |
| **Reference Standard and target condition** |
| plethysmograph |

## Phase 2: Draw the flowchart for the primary study

***Note:*** *It is to evaluate the published flow diagram for the primary study or draw one if none is reported or if the published diagram is not adequate. The flowchart will facilitate judgment of risk of bias and should provide information on the method of patient recruitment (e.g. based on a consecutive series of patients with specific symptoms suspected of having the target condition, or of cases and controls ), the test execution order and the number of patients submitted to the index test and the reference standard. A hand-drawn diagram is sufficient as this step need not be reported as part of the QUADAS-2 assessment.*

| |
|---|
| |

## Phase 3: Risk of bias and applicability judgment

## DOMAIN 1: PATIENT SELECTION

| Describe the patients included (prior testing, presentation, intended use of the index test, and clinical setting | | | |
|---|---|---|---|
| **signaling issues** | **YES** | **NO** | **UNCERTAIN** |
| Were patients recruited consecutively or through random sampling? | | X | |
| Was case-control design avoided? | X | | |
| Did the study avoid inappropriate exclusions? | X | | |

| 1) Risk of bias | LOW | HIGH | UNCERTAIN |
|---|---|---|---|
| Could patient selection have introduced biases?<br>SAMPLE CHARACTERISTICS WAS NOT REPORTED | | X | |
| **2) Concerns related to applicability** | **LOW** | **HIGH** | **UNCERTAIN** |
| Is there a concern that the patients included do not match the research question?<br>SAMPLE CHARACTERISTICS WAS NOT REPORTED | | X | |

**DOMAIN 2: INDEX TEST(S)** (if more than one index test is used, one must be completed for each test)

| Describe the patients included (prior testing, presentation, intended use of the index test, and clinical setting | | | |
|---|---|---|---|
| **signaling issues** | **YES** | **NO** | **UNCERTAIN** |
| Were the index test results interpreted without knowledge of the reference standard results? | | X | |
| Was a positivity threshold used? Was it pre-specified? | | X | |
| **1) Risk of bias** | **LOW** | **HIGH** | **UNCERTAIN** |
| Could the conduct or interpretation of the index test have introduced biases?<br>THE PLETHISMOGRAPH WAS EVALUATED FIRST | | X | |
| **2) Concerns related to applicability** | **LOW** | **HIGH** | **UNCERTAIN** |
| Is there a concern that the index test, its conduct or interpretation differs from the review research question?<br>THE REVIEW SURVEY QUESTION IS THE %F | X | | |

**DOMAIN 3: REFERENCE STANDARD**

| Describe the patients included (prior testing, presentation, intended use of the index test, and clinical setting | | | |
|---|---|---|---|
| **signaling issues** | **YES** | **NO** | **UNCERTAIN** |
| Did the reference standard likely correctly classify the target condition? | X | | |
| Were the reference standard results interpreted without knowledge of the index test results? | X | | |
| **1) Risk of bias** | **LOW** | **HIGH** | **UNCERTAIN** |
| Could the reference standard, its conduct or interpretation of the index test have introduced biases? | X | | |
| **2) Concerns related to applicability** | **LOW** | **HIGH** | **UNCERTAIN** |
| Is there a concern that the target condition defined by the reference standard does not match the research question? | X | | |

**DOMAIN 4: TIME FLOW**

| Please describe any patient who did not receive the index test(s) and/or the reference standard or who were excluded from the 2 x 2 table (refer to the Flowchart)<br>Describe the time lag and any intervention between the index test(s) and the reference standard | | | |
|---|---|---|---|
| **signaling issues** | **YES** | **NO** | **UNCERTAIN** |
| 1. There was an appropriate time interval between the application of the index test(s) and the reference standard | X | | |
| 2. Did all patients receive the reference standard? | X | | |
| 3. Did the patients receive the same reference standard? | X | | |
| 4. Were all patients included in the analyses? | X | | |
| **1) Risk of bias** | **LOW** | **HIGH** | **UNCERTAIN** |
| Could patient flow have introduced bias? | X | | |

## QUADAS-2

| | |
|---|---|
| **TITLE** | Agreement between ultrasound and dual-energy X-ray absorptiometry in assessing percentage body fat in college-aged adults |
| **AUTHOR** | Kelly E. Johnson1,2, Brian Miller1, Judith A. Juvancic-Heltzel1, Sarah E. Agnor1, Dana L. Kiger1, Rachele M. Kappler1 and Ronald Otterstetter |
| **OBJECTIVE** | To employ a cross-over design to investigate the agreement between ULTRA using a seven-site methodology and DXA in assessing body fat (%BF) in a normal-weight sample of college-age men and women |

### PHASE 1: Formulate the review research question

***Note:*** *You should report your systematic review question in terms of patients, index test(s) and reference standard, and target condition. As the accuracy of a test can depend on where the diagnostic pathway will be used; describe the patients in terms of setup, intended use of the index test, patient presentation, and previous tests.*

| **Patients: (clinical setting, intended use of index test, presentation, previous testing)** |
|---|
| N=84 Caucasian adults (35 men and 49 women) with normal weight by BMI.<br>The 2 measurements in this sequence (DEXA and BM)<br>All were evaluated by the index test and the reference standard |
| **Index test(s)** |
| BM |
| **Reference Standard and target condition** |
| DEXA |

### Phase 2: Draw the flowchart for the primary study

***Note:*** *It is to evaluate the published flow diagram for the primary study or draw one if none is reported or if the published diagram is not adequate. The flowchart will facilitate judgment of risk of bias and should provide information on the method of patient recruitment (e.g. based on a consecutive series of patients with specific symptoms suspected of having the target condition, or of cases and controls ), the test execution order and the number of patients submitted to the index test and the reference standard. A hand-drawn diagram is sufficient as this step need not be reported as part of the QUADAS-2 assessment.*

| |
|---|
| |

### Phase 3: Risk of bias and applicability judgment

### DOMAIN 1: PATIENT SELECTION

| Describe the patients included (prior testing, presentation, intended use of the index test, and clinical setting | | | |
|---|---|---|---|
| **signaling issues** | **YES** | **NO** | **UNCERTAIN** |
| Were patients recruited consecutively or through random sampling? | | X | |
| Was case-control design avoided? | X | | |
| Did the study avoid inappropriate exclusions?<br>There were no overweight or obese people in the sample. | | X | |
| **1) Risk of bias** | **LOW** | **HIGH** | **UNCERTAIN** |
| Could patient selection have introduced biases? | | X | |

| WHEN INDIVIDUALS WITH NORMAL WEIGHT BY BMI WERE SELECTED | | | |
|---|---|---|---|
| **2) Concerns related to applicability** | **LOW** | **HIGH** | **UNCERTAIN** |
| Is there a concern that the patients included do not match the research question?<br>WHEN INDIVIDUALS WITH NORMAL WEIGHT BY BMI WERE SELECTED | | X | |

## DOMAIN 2: INDEX TEST(S) (if more than one index test is used, one must be completed for each test)

| Describe the patients included (prior testing, presentation, intended use of the index test, and clinical setting | | | |
|---|---|---|---|
| **signaling issues** | **YES** | **NO** | **UNCERTAIN** |
| Were the index test results interpreted without knowledge of the reference standard results? | | X | |
| Was a positivity threshold used? Was it pre-specified? | | X | |
| **1) Risk of bias** | **LOW** | **HIGH** | **UNCERTAIN** |
| Could the conduct or interpretation of the index test have introduced biases?<br>DEXA WAS EVALUATED FIRST | | X | |
| **2) Concerns related to applicability** | **LOW** | **HIGH** | **UNCERTAIN** |
| Is there a concern that the index test, its conduct or interpretation differs from the review research question?<br>BM EVALUATED THE %G | X | | |

## DOMAIN 3: REFERENCE STANDARD

| Describe the patients included (prior testing, presentation, intended use of the index test, and clinical setting | | | |
|---|---|---|---|
| **signaling issues** | **YES** | **NO** | **UNCERTAIN** |
| Did the reference standard likely correctly classify the target condition? | X | | |
| Were the reference standard results interpreted without knowledge of the index test results? | X | | |
| **1) Risk of bias** | **LOW** | **HIGH** | **UNCERTAIN** |
| Could the reference standard, its conduct or interpretation of the index test have introduced biases? | X | | |
| **2) Concerns related to applicability** | **LOW** | **HIGH** | **UNCERTAIN** |
| Is there a concern that the target condition defined by the reference standard does not match the research question? | X | | |

## DOMAIN 4: TIME FLOW

| Please describe any patient who did not receive the index test(s) and/or the reference standard or who were excluded from the 2 x 2 table (refer to the Flowchart)<br>Describe the time lag and any intervention between the index test(s) and the reference standard | | | |
|---|---|---|---|
| **signaling issues** | **YES** | **NO** | **UNCERTAIN** |
| 1. There was an appropriate time interval between the application of the index test(s) and the reference standard | | | X |
| 2. Did all patients receive the reference standard? | X | | |
| 3. Did the patients receive the same reference standard? | X | | |
| 4. Were all patients included in the analyses? | X | | |
| **1) Risk of bias** | **LOW** | **HIGH** | **UNCERTAIN** |
| Could patient flow have introduced bias? | | | X |

**QUADAS-2**

| | |
|---|---|
| **TITLE** | Validity of the Portable Ultrasound BodyMetrix — BX-2000 for Measuring Body Fat Percentage<br><br>Validity of BodyMetrix Portable Ultrasound BX-2000 for Body Fat Percentage Measurement |
| **AUTHOR** | KANG ET AL., 2020 |
| **OBJECTIVE** | Systemically investigate possible errors in different protocols and demonstrate the validity of %BF between each different protocol in BodyMetrix compared to the criterion method (ie DEXA) through further statistical analysis (ie Equivalence Test). |

## PHASE 1: Formulate the review research question

***Note:*** *You should report your systematic review question in terms of patients, index test(s) and reference standard, and target condition. As the accuracy of a test can depend on where the diagnostic pathway will be used; describe the patients in terms of setup, intended use of the index test, patient presentation, and previous tests.*

| **Patients: (clinical setting, intended use of index test, presentation, previous testing)** |
|---|
| • N= 105 university students;<br>• Non-randomized sample;<br>• Age: 20.01 ± 2.11 years; Mass: 73.26 ± 13.6 kg; Height: 1.72 ± 0.10 m; BMI: 23.91 ± 3.77 Kg/m $^2$;<br>• Exclusion: Does not report;<br>• The 04 measurements were in sequence: height and weight, BMI, %F DEXA and BM.<br>• It does not inform data collection time and days. |
| **Index test(s)** |
| bodymetrix |
| **Reference Standard and target condition** |
| DEXA |

## Phase 2: Draw the flowchart for the primary study

***Note:*** *It is to evaluate the published flow diagram for the primary study or draw one if none is reported or if the published diagram is not adequate. The flowchart will facilitate judgment of risk of bias and should provide information on the method of patient recruitment (e.g. based on a consecutive series of patients with specific symptoms suspected of having the target condition, or of cases and controls ), the test execution order and the number of patients submitted to the index test and the reference standard. A hand-drawn diagram is sufficient as this step need not be reported as part of the QUADAS-2 assessment.*

| |
|---|
| |

## Phase 3: Risk of bias and applicability judgment

## DOMAIN 1: PATIENT SELECTION

| |
|---|
| Describe the patients included (prior testing, presentation, intended use of the index test, and clinical setting |

| QUADAS-2 | | | |
|---|---|---|---|
| **signaling issues** | **YES** | **NO** | **UNCERTAIN** |
| Were patients recruited consecutively or through random sampling? | | X | |
| **Was case-control design avoided?** | **X** | | |
| Did the study avoid inappropriate exclusions? The study does not report | | | x |
| **1) Risk of bias** | **LOW** | **HIGH** | **UNCERTAIN** |
| Could patient selection have introduced biases? | | X | |
| **2) Concerns related to applicability** | **LOW** | **HIGH** | **UNCERTAIN** |
| Is there a concern that the patients included do not match the research question? | X | | |

**DOMAIN 2: INDEX TEST(S)** (if more than one index test is used, one must be completed for each test)

| |
|---|
| Describe the patients included (prior testing, presentation, intended use of the index test, and clinical setting<br>Height and weight, BMI, %F DEXA, and BM. |

| **signaling issues** | **YES** | **NO** | **UNCERTAIN** |
|---|---|---|---|
| Were the index test results interpreted without knowledge of the reference standard results? | | X | |
| **Was a positivity threshold used? Was it pre-specified?** | | **X** | |
| **1) Risk of bias** | **LOW** | **HIGH** | **UNCERTAIN** |
| Could the conduct or interpretation of the index test have introduced biases? Assessed the %F | | X | |
| **2) Concerns related to applicability** | **LOW** | **HIGH** | **UNCERTAIN** |
| Is there a concern that the index test, its conduct or interpretation differs from the review research question? Does Bodymetrix evaluate the % fat? | X | | |

**DOMAIN 3: REFERENCE STANDARD**

| |
|---|
| Describe the patients included (prior testing, presentation, intended use of the index test, and clinical setting |

| **signaling issues** | **YES** | **NO** | **UNCERTAIN** |
|---|---|---|---|
| Did the reference standard likely correctly classify the target condition? | X | | |
| **Were the reference standard results interpreted without knowledge of the index test results?** | **X** | | |
| **1) Risk of bias** | **LOW** | **HIGH** | **UNCERTAIN** |
| Could the reference standard, its conduct or interpretation of the index test have introduced biases? | X | | |
| **2) Concerns related to applicability** | **LOW** | **HIGH** | **UNCERTAIN** |
| Is there a concern that the target condition defined by the reference standard does not match the research question? | X | | |

**DOMAIN 4: TIME FLOW**

Please describe any patient who did not receive the index test(s) and/or the reference standard or who were excluded from the 2 x 2 table (refer to the Flowchart)
It does not inform data collection time and days.

| **signaling issues** | **YES** | **NO** | **UNCERTAIN** |
|---|---|---|---|
| 1. There was an appropriate time interval between the application of the index test(s) and the reference standard | | | X |
| 2. Did all patients receive the reference standard? | X | | |
| 3. Did the patients receive the same reference standard? | X | | |
| 4. Were all patients included in the analyses? | X | | |
| **1) Risk of bias** | **LOW** | **HIGH** | **UNCERTAIN** |
| Could patient flow have introduced bias? | | | X |

# QUADAS-2

| | |
|---|---|
| **TITLE** | Validity of a portable computer-based ultrasound system for estimating adipose tissue in female gymnasts |
| **AUTHOR** | Jeremy P. Loenneke1, Jeremy T. Barnes2, Jason D. Wagganer2 and Thomas J. Pujo |
| **OBJECTIVE** | Determine the validity of a portable ultrasound instrument for estimating adipose tissue (AT%) compared to dual-energy X-ray absorptiometry (DXA) in female collegiate gymnasts |

## PHASE 1: Formulate the review research question

***Note:*** *You should report your systematic review question in terms of patients, index test(s) and reference standard, and target condition. As the accuracy of a test can depend on where the diagnostic pathway will be used; describe the patients in terms of setup, intended use of the index test, patient presentation, and previous tests.*

| **Patients: (clinical setting, intended use of index test, presentation, previous testing)** |
|---|
| N=13 female gymnasts.<br>The 2 measurements in this sequence (BM and DEXA)<br>All were evaluated by the index test and the reference standard |
| **Index test(s)** |
| BM |
| **Reference Standard and target condition** |
| DEXA |

## Phase 2: Draw the flowchart for the primary study

***Note:*** *It is to evaluate the published flow diagram for the primary study or draw one if none is reported or if the published diagram is not adequate. The flowchart will facilitate judgment of risk of bias and should provide information on the method of patient recruitment (e.g. based on a consecutive series of patients with specific symptoms suspected of having the target condition, or of cases and controls ), the test execution order and the number of patients submitted to the index test and the reference standard. A hand-drawn diagram is sufficient as this step need not be reported as part of the QUADAS-2 assessment.*

| |
|---|
| |

## Phase 3: Risk of bias and applicability judgment

## DOMAIN 1: PATIENT SELECTION

| Describe the patients included (prior testing, presentation, intended use of the index test, and clinical setting | | | |
|---|---|---|---|
| **signaling issues** | **YES** | **NO** | **UNCERTAIN** |
| Were patients recruited consecutively or through random sampling? | | X | |
| Was case-control design avoided? | X | | |
| Did the study avoid inappropriate exclusions? | X | | |
| **1) Risk of bias** | **LOW** | **HIGH** | **UNCERTAIN** |
| Could patient selection have introduced biases?<br>GYMNASTS WITH LOW %F | | X | |
| **2) Concerns related to applicability** | **LOW** | **HIGH** | **UNCERTAIN** |

| | | | |
|---|---|---|---|
| Is there a concern that the patients included do not match the research question?<br>GYMNASTS WITH LOW %F | | X | |

## DOMAIN 2: INDEX TEST(S) (if more than one index test is used, one must be completed for each test)

| Describe the patients included (prior testing, presentation, intended use of the index test, and clinical setting | | | |
|---|---|---|---|
| **signaling issues** | **YES** | **NO** | **UNCERTAIN** |
| Were the index test results interpreted without knowledge of the reference standard results? | X | | |
| Was a positivity threshold used? Was it pre-specified? | | X | |
| **1) Risk of bias** | **LOW** | **HIGH** | **UNCERTAIN** |
| Could the conduct or interpretation of the index test have introduced biases? | X | | |
| **2) Concerns related to applicability** | **LOW** | **HIGH** | **UNCERTAIN** |
| Is there a concern that the index test, its conduct or interpretation differs from the review research question?<br>BM EVALUATES THE %F | X | | |

## DOMAIN 3: REFERENCE STANDARD

| Describe the patients included (prior testing, presentation, intended use of the index test, and clinical setting | | | |
|---|---|---|---|
| **signaling issues** | **YES** | **NO** | **UNCERTAIN** |
| Did the reference standard likely correctly classify the target condition? | X | | |
| Were the reference standard results interpreted without knowledge of the index test results? | X | | |
| **1) Risk of bias** | **LOW** | **HIGH** | **UNCERTAIN** |
| Could the reference standard, its conduct or interpretation of the index test have introduced biases? | X | | |
| **2) Concerns related to applicability** | **LOW** | **HIGH** | **UNCERTAIN** |
| Is there a concern that the target condition defined by the reference standard does not match the research question?<br>EVALUATING THE %F | X | | |

## DOMAIN 4: TIME FLOW

| Please describe any patient who did not receive the index test(s) and/or the reference standard or who were excluded from the 2 x 2 table (refer to the Flowchart)<br>Describe the time lag and any intervention between the index test(s) and the reference standard | | | |
|---|---|---|---|
| **signaling issues** | **YES** | **NO** | **UNCERTAIN** |
| 1. There was an appropriate time interval between the application of the index test(s) and the reference standard | | | X |
| 2. Did all patients receive the reference standard? | X | | |
| 3. Did the patients receive the same reference standard? | X | | |
| 4. Were all patients included in the analyses? | X | | |
| **1) Risk of bias** | **LOW** | **HIGH** | **UNCERTAIN** |
| Could patient flow have introduced bias? | | | X |

**QUADAS-2**

| | |
|---|---|
| **TITLE** | Fat Percentage Evaluation Through Portable Ultrasound in Adolescents: A Comparison with Dual-energy X-ray Absorptiometry |
| **AUTHOR** | wagner luis Ripka , Pedro Miguel Gewehr , Leandra Ulbricht |
| **OBJECTIVE** | Analyzing the performance of portable US, in comparison with dual-energy X-ray absorptiometry (DXA) reference method, in specific equations for predicting fat percentage in male adolescents |

## PHASE 1: Formulate the review research question

***Note:*** *You should report your systematic review question in terms of patients, index test(s) and reference standard, and target condition. As the accuracy of a test can depend on where the diagnostic pathway will be used; describe the patients in terms of setup, intended use of the index test, patient presentation, and previous tests.*

| **Patients: (clinical setting, intended use of index test, presentation, previous testing)** |
|---|
| N=143 male adolescents, aged 12 to 17 years, with an average BMI of 21.16, performed measurements with BM and DEXA, in this sequence. |
| **Index test(s)** |
| BM |
| **Reference Standard and target condition** |
| DEXA |

## Phase 2: Draw the flowchart for the primary study

***Note:*** *It is to evaluate the published flow diagram for the primary study or draw one if none is reported or if the published diagram is not adequate. The flowchart will facilitate judgment of risk of bias and should provide information on the method of patient recruitment (e.g. based on a consecutive series of patients with specific symptoms suspected of having the target condition, or of cases and controls ), the test execution order and the number of patients submitted to the index test and the reference standard. A hand-drawn diagram is sufficient as this step need not be reported as part of the QUADAS-2 assessment.*

| |
|---|
| |

## Phase 3: Risk of bias and applicability judgment

## DOMAIN 1: PATIENT SELECTION

| Describe the patients included (prior testing, presentation, intended use of the index test, and clinical setting | | | |
|---|---|---|---|
| **signaling issues** | **YES** | **NO** | **UNCERTAIN** |
| Were patients recruited consecutively or through random sampling? | | X | |
| Was case-control design avoided? | X | | |
| Did the study avoid inappropriate exclusions? | X | | |
| **1) Risk of bias** | **LOW** | **HIGH** | **UNCERTAIN** |
| Could patient selection have introduced biases? MALE ADOLESCENTS WITH A BMI OF 21 | | X | |
| **2) Concerns related to applicability** | **LOW** | **HIGH** | **UNCERTAIN** |
| Is there a concern that the patients included do not match the research question? | | X | |

| | | | |
|---|---|---|---|
| MALE ADOLESCENTS WITH A BMI OF 21 | | | |

## DOMAIN 2: INDEX TEST(S) (if more than one index test is used, one must be completed for each test)

| Describe the patients included (prior testing, presentation, intended use of the index test, and clinical setting | | | |
|---|---|---|---|
| **signaling issues** | **YES** | **NO** | **UNCERTAIN** |
| Were the index test results interpreted without knowledge of the reference standard results? | X | | |
| Was a positivity threshold used? Was it pre-specified? | | X | |
| **1) Risk of bias** | **LOW** | **HIGH** | **UNCERTAIN** |
| Could the conduct or interpretation of the index test have introduced biases? | X | | |
| **2) Concerns related to applicability** | **LOW** | **HIGH** | **UNCERTAIN** |
| Is there a concern that the index test, its conduct or interpretation differs from the review research question?<br>BM EVALUATES THE %F | X | | |

## DOMAIN 3: REFERENCE STANDARD

| Describe the patients included (prior testing, presentation, intended use of the index test, and clinical setting | | | |
|---|---|---|---|
| **signaling issues** | **YES** | **NO** | **UNCERTAIN** |
| Did the reference standard likely correctly classify the target condition? | X | | |
| Were the reference standard results interpreted without knowledge of the index test results? | X | | |
| **1) Risk of bias** | **LOW** | **HIGH** | **UNCERTAIN** |
| Could the reference standard, its conduct or interpretation of the index test have introduced biases? | X | | |
| **2) Concerns related to applicability** | **LOW** | **HIGH** | **UNCERTAIN** |
| Is there a concern that the target condition defined by the reference standard does not match the research question?<br>EVALUATING THE %F | X | | |

## DOMAIN 4: TIME FLOW

| Please describe any patient who did not receive the index test(s) and/or the reference standard or who were excluded from the 2 x 2 table (refer to the Flowchart)<br>Describe the time lag and any intervention between the index test(s) and the reference standard | | | |
|---|---|---|---|
| **signaling issues** | **YES** | **NO** | **UNCERTAIN** |
| 1. There was an appropriate time interval between the application of the index test(s) and the reference standard | | | X |
| 2. Did all patients receive the reference standard? | X | | |
| 3. Did the patients receive the same reference standard? | X | | |
| 4. Were all patients included in the analyses? | X | | |
| **1) Risk of bias** | **LOW** | **HIGH** | **UNCERTAIN** |
| Could patient flow have introduced bias? | | | X |

# QUADAS-2

| | |
|---|---|
| **TITLE** | Portable A-Mode Ultrasound for Body Composition Assessment in Adolescents |
| **AUTHOR** | wagner luis Ripka , Leandra Ulbricht , Lucas Menghin , Pedro Miguel Gewehr |
| **OBJECTIVE** | Assess the agreement of portable US with a reference standard method, dual-energy x-ray absorptiometry (DXA), for body fat percentage (BF%) in adolescents and verify whether the use of a new mathematical model, based on the anatomical thickness obtained by the US, is capable of improving BF% prediction. |

## PHASE 1: Formulate the review research question

***Note:*** *You should report your systematic review question in terms of patients, index test(s) and reference standard, and target condition. As the accuracy of a test can depend on where the diagnostic pathway will be used; describe the patients in terms of setup, intended use of the index test, patient presentation, and previous tests.*

| **Patients: (clinical setting, intended use of index test, presentation, previous testing)** |
|---|
| N=105 adolescents, 71 males, and 34 females, aged 12 to 17 years, with an average BMI of 20, performed measurements with DEXA and BM, in this sequence. |
| **Index test(s)** |
| BM |
| **Reference Standard and target condition** |
| DEXA |

## Phase 2: Draw the flowchart for the primary study

***Note:*** *It is to evaluate the published flow diagram for the primary study or draw one if none is reported or if the published diagram is not adequate. The flowchart will facilitate judgment of risk of bias and should provide information on the method of patient recruitment (e.g. based on a consecutive series of patients with specific symptoms suspected of having the target condition, or of cases and controls ), the test execution order and the number of patients submitted to the index test and the reference standard. A hand-drawn diagram is sufficient as this step need not be reported as part of the QUADAS-2 assessment.*

| |
|---|
| |

## Phase 3: Risk of bias and applicability judgment

## DOMAIN 1: PATIENT SELECTION

| Describe the patients included (prior testing, presentation, intended use of the index test, and clinical setting | | | |
|---|---|---|---|
| **signaling issues** | **YES** | **NO** | **UNCERTAIN** |
| Were patients recruited consecutively or through random sampling? | | X | |
| Was case-control design avoided? | X | | |
| Did the study avoid inappropriate exclusions? | X | | |
| **1) Risk of bias** | **LOW** | **HIGH** | **UNCERTAIN** |
| Could patient selection have introduced biases? ADOLESCENTS WITH A BMI OF 20 | | X | |

| 2) Concerns related to applicability | LOW | HIGH | UNCERTAIN |
|---|---|---|---|
| Is there a concern that the patients included do not match the research question?<br>ADOLESCENTS WITH A BMI OF 20 | | x | |

## DOMAIN 2: INDEX TEST(S) (if more than one index test is used, one must be completed for each test)

| Describe the patients included (prior testing, presentation, intended use of the index test, and clinical setting | | | |
|---|---|---|---|
| **signaling issues** | **YES** | **NO** | **UNCERTAIN** |
| Were the index test results interpreted without knowledge of the reference standard results? | | X | |
| Was a positivity threshold used? Was it pre-specified? | | X | |
| **1) Risk of bias** | **LOW** | **HIGH** | **UNCERTAIN** |
| Could the conduct or interpretation of the index test have introduced biases? | | X | |
| **2) Concerns related to applicability** | **LOW** | **HIGH** | **UNCERTAIN** |
| Is there a concern that the index test, its conduct or interpretation differs from the review research question?<br>BM EVALUATES THE %F | X | | |

## DOMAIN 3: REFERENCE STANDARD

| Describe the patients included (prior testing, presentation, intended use of the index test, and clinical setting | | | |
|---|---|---|---|
| **signaling issues** | **YES** | **NO** | **UNCERTAIN** |
| Did the reference standard likely correctly classify the target condition? | X | | |
| Were the reference standard results interpreted without knowledge of the index test results? | X | | |
| **1) Risk of bias** | **LOW** | **HIGH** | **UNCERTAIN** |
| Could the reference standard, its conduct or interpretation of the index test have introduced biases? | X | | |
| **2) Concerns related to applicability** | **LOW** | **HIGH** | **UNCERTAIN** |
| Is there a concern that the target condition defined by the reference standard does not match the research question?<br>EVALUATING THE %F | X | | |

## DOMAIN 4: TIME FLOW

| Please describe any patient who did not receive the index test(s) and/or the reference standard or who were excluded from the 2 x 2 table (refer to the Flowchart)<br>Describe the time lag and any intervention between the index test(s) and the reference standard | | | |
|---|---|---|---|
| **signaling issues** | **YES** | **NO** | **UNCERTAIN** |
| 1. There was an appropriate time interval between the application of the index test(s) and the reference standard | | | X |
| 2. Did all patients receive the reference standard? | X | | |
| 3. Did the patients receive the same reference standard? | X | | |
| 4. Were all patients included in the analyses? | X | | |
| **1) Risk of bias** | **LOW** | **HIGH** | **UNCERTAIN** |
| Could patient flow have introduced bias? | | | X |

# QUADAS-2

| | |
|---|---|
| **TITLE** | Comparison of amplitude-mode ultrasound versus air displacement plethysmography for assessing body composition changes following participation in a structured weight-loss program in women |
| **AUTHOR** | Brad J. Schoenfeld1, Alan A. Aragon2, Jordan Moon3, James W. Krieger4 and Gul Tiryaki-Sonmez1 |
| **OBJECTIVE** | Compare body composition changes as measured by A-mode ultrasound (US) versus a criterion densitometry-based measure, air displacement plethysmography (ADP) |

## PHASE 1: Formulate the review research question

***Note:*** *You should report your systematic review question in terms of patients, index test(s) and reference standard, and target condition. As the accuracy of a test can depend on where the diagnostic pathway will be used; describe the patients in terms of setup, intended use of the index test, patient presentation, and previous tests.*

| **Patients: (clinical setting, intended use of index test, presentation, previous testing)** |
|---|
| N=20 healthy, non-obese women, with a mean BMI of 23, excluded obese subjects from the sample to assess F% and FFM.<br>plethysmograph and BM tests in this sequence |
| **Index test(s)** |
| BM |
| **Reference Standard and target condition** |
| plethysmograph |

## Phase 2: Draw the flowchart for the primary study

***Note:*** *It is to evaluate the published flow diagram for the primary study or draw one if none is reported or if the published diagram is not adequate. The flowchart will facilitate judgment of risk of bias and should provide information on the method of patient recruitment (e.g. based on a consecutive series of patients with specific symptoms suspected of having the target condition, or of cases and controls ), the test execution order and the number of patients submitted to the index test and the reference standard. A hand-drawn diagram is sufficient as this step need not be reported as part of the QUADAS-2 assessment.*

| |
|---|
| |

## Phase 3: Risk of bias and applicability judgment

## DOMAIN 1: PATIENT SELECTION

| Describe the patients included (prior testing, presentation, intended use of the index test, and clinical setting | | | |
|---|---|---|---|
| **signaling issues** | **YES** | **NO** | **UNCERTAIN** |
| Were patients recruited consecutively or through random sampling? | | X | |
| Was case-control design avoided? | X | | |
| Did the study avoid inappropriate exclusions? | | X | |
| **1) Risk of bias** | **LOW** | **HIGH** | **UNCERTAIN** |
| Could patient selection have introduced biases?<br>WITHDRAWN FROM THE SAMPLE OBESE | | X | |

| 2) Concerns related to applicability | LOW | HIGH | UNCERTAIN |
|---|---|---|---|
| Is there a concern that the patients included do not match the research question?<br>WITHDRAWN FROM THE SAMPLE OBESE | | x | |

## DOMAIN 2: INDEX TEST(S) (if more than one index test is used, one must be completed for each test)

| Describe the patients included (prior testing, presentation, intended use of the index test, and clinical setting | | | |
|---|---|---|---|
| **signaling issues** | **YES** | **NO** | **UNCERTAIN** |
| Were the index test results interpreted without knowledge of the reference standard results? | | x | |
| Was a positivity threshold used? Was it pre-specified? | | X | |
| **1) Risk of bias** | **LOW** | **HIGH** | **UNCERTAIN** |
| Could the conduct or interpretation of the index test have introduced biases? | | x | |
| **2) Concerns related to applicability** | **LOW** | **HIGH** | **UNCERTAIN** |
| Is there a concern that the index test, its conduct or interpretation differs from the review research question?<br>BM EVALUATES THE %GEA MLG | x | | |

## DOMAIN 3: REFERENCE STANDARD

| Describe the patients included (prior testing, presentation, intended use of the index test, and clinical setting | | | |
|---|---|---|---|
| **signaling issues** | **YES** | **NO** | **UNCERTAIN** |
| Did the reference standard likely correctly classify the target condition? | X | | |
| Were the reference standard results interpreted without knowledge of the index test results? | X | | |
| **1) Risk of bias** | **LOW** | **HIGH** | **UNCERTAIN** |
| Could the reference standard, its conduct or interpretation of the index test have introduced biases? | X | | |
| **2) Concerns related to applicability** | **LOW** | **HIGH** | **UNCERTAIN** |
| Is there a concern that the target condition defined by the reference standard does not match the research question?<br>EVALUATING THE %GEA MLG | X | | |

## DOMAIN 4: TIME FLOW

| Please describe any patient who did not receive the index test(s) and/or the reference standard or who were excluded from the 2 x 2 table (refer to the Flowchart)<br>Describe the time lag and any intervention between the index test(s) and the reference standard | | | |
|---|---|---|---|
| **signaling issues** | **YES** | **NO** | **UNCERTAIN** |
| 1. There was an appropriate time interval between the application of the index test(s) and the reference standard | | | X |
| 2. Did all patients receive the reference standard? | x | | |
| 3. Did the patients receive the same reference standard? | X | | |
| 4. Were all patients included in the analyses? | X | | |
| **1) Risk of bias** | **LOW** | **HIGH** | **UNCERTAIN** |
| Could patient flow have introduced bias? | x | | |

## QUADAS-2

| | |
|---|---|
| **TITLE** | Test-retest reliability and validity of body composition methods in adults<br><br>retest reliability and validity of body composition methods in adults |
| **AUTHOR** | TOTOSY DE ZEPETNEK et al., 2021 |
| **OBJECTIVE** | to evaluate the test-retest reliability and validity of three body composition measurement devices. |

### PHASE 1: Formulate the review research question

***Note:*** *You should report your systematic review question in terms of patients, index test(s) and reference standard, and target condition. As the accuracy of a test can depend on where the diagnostic pathway will be used; describe the patients in terms of setup, intended use of the index test, patient presentation, and previous tests.*

| **Patients: (clinical setting, intended use of index test, presentation, previous testing)** |
|---|
| • participants ages 18-65 were recruited through word of mouth, social media, and flyers posted on and around the Ryerson campus University (Toronto, ON, Canada) ;<br>• N= 49 adults – 33 (F) and 16 (M), volunteers;<br>• Age: 31.4 ± 10.7; Weight: 68.0 ± 12.9; Height: 1.69 ± 0.8; BMI: 23.5 ± 3.0; %F: 24.7 ± 7.2);<br>• Rated %G;<br>• Exclusion criteria included anyone unable to complete all body composition assessments (eg, non-ambulatory) and/or was pregnant or breastfeeding.<br>• Non-randomized sample;<br>• An initial screening took place via email to determine study eligibility.<br>• The 03 measurements were in sequence: BM, Bioimpedance, and Plethysmograph; In a single 3-hour session, written informed consent was obtained;<br>• % BF was evaluated using the reference PDA (BOD POD®, Life Measurement Instruments );<br>• three body composition assessment devices were used to estimate %BF in duplicate;<br>• (1) seven-site skinfold thickness using Lange calipers (Cambridge Scientific Inc.), (2) seven-site skinfold thickness using BodyMetrix BX-2000 A-mode ultrasound ( BodyMetrix ™, Intelametrix ), and ( 3) Full body 3DPS via Fit3D Proscanner ©, Fit 3D Inc.)<br>• All measurements were collected from each participant on the same day, except for the plethysmograph, which was collected as soon as possible on a different day. |
| **Index test(s)** |
| bodymetrix |
| **Reference Standard and target condition** |
| plethysmograph |

### Phase 2: Draw the flowchart for the primary study

***Note:*** *It is to evaluate the published flow diagram for the primary study or draw one if none is reported or if the published diagram is not adequate. The flowchart will facilitate judgment of risk of bias and should provide information on the method of patient recruitment (e.g. based on a consecutive series of patients with specific symptoms suspected of having the target condition, or of cases and controls ), the test execution order and the number of*

*patients submitted to the index test and the reference standard. A hand-drawn diagram is sufficient as this step need not be reported as part of the QUADAS-2 assessment.*

## Phase 3: Risk of bias and applicability judgment

### DOMAIN 1: PATIENT SELECTION

| Describe the patients included (prior testing, presentation, intended use of the index test, and clinical setting | | | |
|---|---|---|---|
| **signaling issues** | **YES** | **NO** | **UNCERTAIN** |
| Were patients recruited consecutively or through random sampling? email triage | | X | |
| **Was case-control design avoided?** | **X** | | |
| Did the study avoid inappropriate exclusions? | X | | |
| **1) Risk of bias** | **LOW** | **HIGH** | **UNCERTAIN** |
| Could patient selection have introduced biases? Middle-aged, normal-weight patients | | X | |
| **2) Concerns related to applicability** | **LOW** | **HIGH** | **UNCERTAIN** |
| Is there a concern that the patients included do not match the research question? | X | | |

### DOMAIN 2: INDEX TEST(S) (if more than one index test is used, one must be completed for each test)

| Describe the patients included (prior testing, presentation, intended use of the index test, and clinical setting<br>Plethysmograph, DC, BM, and Fit3D | | | |
|---|---|---|---|
| **signaling issues** | **YES** | **NO** | **UNCERTAIN** |
| Were the index test results interpreted without knowledge of the reference standard results? | | X | |
| **Was a positivity threshold used? Was it pre-specified?** | | **X** | |
| **1) Risk of bias** | **LOW** | **HIGH** | **UNCERTAIN** |
| Could the conduct or interpretation of the index test have introduced biases?<br>Evaluated the % fat | | X | |
| **2) Concerns related to applicability** | **LOW** | **HIGH** | **UNCERTAIN** |
| Is there a concern that the index test, its conduct or interpretation differs from the review research question? The Bodymetrix evaluates the % fat | X | | |

### DOMAIN 3: REFERENCE STANDARD

| Describe the patients included (prior testing, presentation, intended use of the index test, and clinical setting | | | |
|---|---|---|---|
| **signaling issues** | **YES** | **NO** | **UNCERTAIN** |
| Did the reference standard likely correctly classify the target condition? | X | | |
| **Were the reference standard results interpreted without knowledge of the index test results?** | **X** | | |
| **1) Risk of bias** | **LOW** | **HIGH** | **UNCERTAIN** |

| | | | |
|---|---|---|---|
| Could the reference standard, its conduct or interpretation of the index test have introduced biases? | X | | |
| **2) Concerns related to applicability** | **LOW** | **HIGH** | **UNCERTAIN** |
| Is there a concern that the target condition defined by the reference standard does not match the research question?<br>The plethysmograph evaluates O% G | X | | |

## DOMAIN 4: TIME FLOW

| | | | |
|---|---|---|---|
| Please describe any patient who did not receive the index test(s) and/or the reference standard or who were excluded from the 2 x 2 table (refer to the Flowchart)<br>All measurements were collected from each participant on the same day<br>In the total sample: 49 | | | |
| **signaling issues** | **YES** | **NO** | **UNCERTAIN** |
| 1. There was an appropriate time interval between the application of the index test(s) and the reference standard | X | | |
| 2. Did all patients receive the reference standard? | X | | |
| 3. Did the patients receive the same reference standard? | X | | |
| 4. Were all patients included in the analyses? | X | | |
| **1) Risk of bias** | **LOW** | **HIGH** | **UNCERTAIN** |
| Could patient flow have introduced bias? | X | | |

# QUADAS-2

| | |
|---|---|
| **TITLE** | Validity and Reliability of A-Mode Ultrasound for Body Composition Assessment of NCAA Division I Athletes |
| **AUTHOR** | Dale R. Wagner*, Dustin L. Cain, Nicolas W. Clark |
| **OBJECTIVE** | This study evaluated the validity and reliability of the BodyMetrix™BX2000 A-mode ultrasound for estimating percent body fat (%BF) in athletes by comparing it to skinfolds and the BOD POD |

## PHASE 1: Formulate the review research question

***Note:*** *You should report your systematic review question in terms of patients, index test(s) and reference standard, and target condition. As the accuracy of a test can depend on where the diagnostic pathway will be used; describe the patients in terms of setup, intended use of the index test, patient presentation, and previous tests.*

| **Patients: (clinical setting, intended use of index test, presentation, previous testing)** |
|---|
| N=45 collegiate athletes 22 men and 23 women<br>Measured by the plethysmograph and then by the BM BUT THEY WERE BLIND TO THE RESULTS<br>Average BMI of 24 |
| **Index test(s)** |
| BM |
| **Reference Standard and target condition** |
| plethysmograph |

## Phase 2: Draw the flowchart for the primary study

***Note:*** *It is to evaluate the published flow diagram for the primary study or draw one if none is reported or if the published diagram is not adequate. The flowchart will facilitate judgment of risk of bias and should provide information on the method of patient recruitment (e.g. based on a consecutive series of patients with specific symptoms suspected of having the target condition, or of cases and controls ), the test execution order and the number of patients submitted to the index test and the reference standard. A hand-drawn diagram is sufficient as this step need not be reported as part of the QUADAS-2 assessment.*

| |
|---|
| |

## Phase 3: Risk of bias and applicability judgment

## DOMAIN 1: PATIENT SELECTION

| Describe the patients included (prior testing, presentation, intended use of the index test, and clinical setting | | | |
|---|---|---|---|
| **signaling issues** | **YES** | **NO** | **UNCERTAIN** |
| Were patients recruited consecutively or through random sampling? | | X | |
| Was case-control design avoided? | X | | |
| Did the study avoid inappropriate exclusions? | X | | |
| **1) Risk of bias** | **LOW** | **HIGH** | **UNCERTAIN** |
| Could patient selection have introduced biases?<br>ATHLETES WITH AVERAGE BMI OF 24 | | X | |
| **2) Concerns related to applicability** | **LOW** | **HIGH** | **UNCERTAIN** |

| | | | |
|---|---|---|---|
| Is there a concern that the patients included do not match the research question?<br>ATHLETES WITH AVERAGE BMI OF 24 | X | | |

## DOMAIN 2: INDEX TEST(S) (if more than one index test is used, one must be completed for each test)

| Describe the patients included (prior testing, presentation, intended use of the index test, and clinical setting | | | |
|---|---|---|---|
| **signaling issues** | **YES** | **NO** | **UNCERTAIN** |
| Were the index test results interpreted without knowledge of the reference standard results? | X | | |
| Was a positivity threshold used? Was it pre-specified? | | X | |
| **1) Risk of bias** | **LOW** | **HIGH** | **UNCERTAIN** |
| Could the conduct or interpretation of the index test have introduced biases? | | X | |
| **2) Concerns related to applicability** | **LOW** | **HIGH** | **UNCERTAIN** |
| Is there a concern that the index test, its conduct or interpretation differs from the review research question?<br>BM EVALUATES THE %F | X | | |

## DOMAIN 3: REFERENCE STANDARD

| Describe the patients included (prior testing, presentation, intended use of the index test, and clinical setting | | | |
|---|---|---|---|
| **signaling issues** | **YES** | **NO** | **UNCERTAIN** |
| Did the reference standard likely correctly classify the target condition? | X | | |
| Were the reference standard results interpreted without knowledge of the index test results? | X | | |
| **1) Risk of bias** | **LOW** | **HIGH** | **UNCERTAIN** |
| Could the reference standard, its conduct or interpretation of the index test have introduced biases? | X | | |
| **2) Concerns related to applicability** | **LOW** | **HIGH** | **UNCERTAIN** |
| Is there a concern that the target condition defined by the reference standard does not match the research question?<br>EVALUATING THE %G | X | | |

## DOMAIN 4: TIME FLOW

| Please describe any patient who did not receive the index test(s) and/or the reference standard or who were excluded from the 2 x 2 table (refer to the Flowchart)<br>Describe the time lag and any intervention between the index test(s) and the reference standard | | | |
|---|---|---|---|
| **signaling issues** | **YES** | **NO** | **UNCERTAIN** |
| 1. There was an appropriate time interval between the application of the index test(s) and the reference standard<br>FOLLOWING | X | | |
| 2. Did all patients receive the reference standard? | X | | |
| 3. Did the patients receive the same reference standard? | X | | |
| 4. Were all patients included in the analyses? | X | | |
| **1) Risk of bias** | **LOW** | **HIGH** | **UNCERTAIN** |
| Could patient flow have introduced bias? | X | | |
