# Supplementary material for: Validity and reliability of portable A-mode ultrasound in measuring body fat percentage: A systematic review with meta-analysis
Source: PLoS One. 2024 Feb 8;19(2):e0292872. doi: 10.1371/journal.pone.0292872 (PMC10852247; doi:10.1371/journal.pone.0292872)
Supplement: S4 File — (ZIP) [file pone.0292872.s005.zip › Statistical analysis supplement/Reliability all studies fixed effect.pdf]

Results

Meta-Analysis

Fixed-Effects Model (k = 12)

	**Estimate**	**se**	**Z**	**p**	**CI Lower Bound**	**CI Upper Bound**
Intercept	0.207	0.513	0.404	0.686	-0.798	1.212
	.	.	.	.	.	.

[3]

Heterogeneity Statistics

**Tau**	**Tau^2**	**I^2**	**H^2**	**R^2**	**df**	**Q**	**p**
0.000	0 (SE= NA)	19.44%	1.241	.	11.000	13.655	0.253

Forest Plot

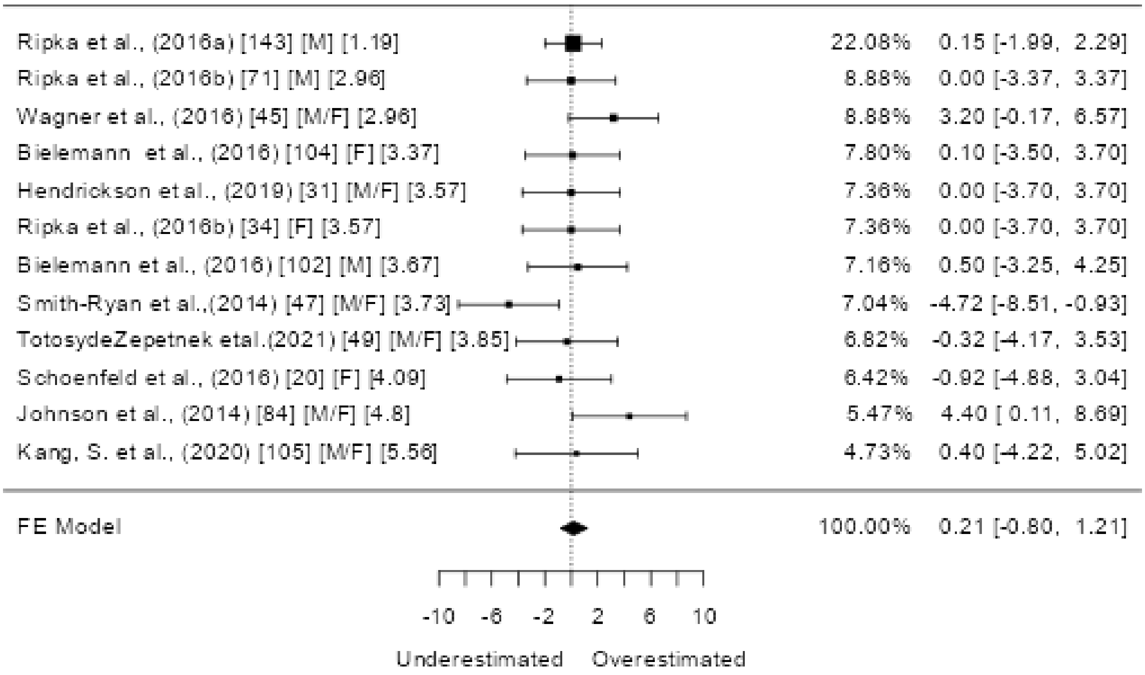

[3]

Publication Bias Assessment

Test Name	value	p
Fail-Safe N	0.000	0.340
Kendalls Tau	-0.093	0.679
Egger's Regression	0.083	0.934

Nota. Fail-safe N Calculation Using the Rosenthal Approach

Funnel Plot

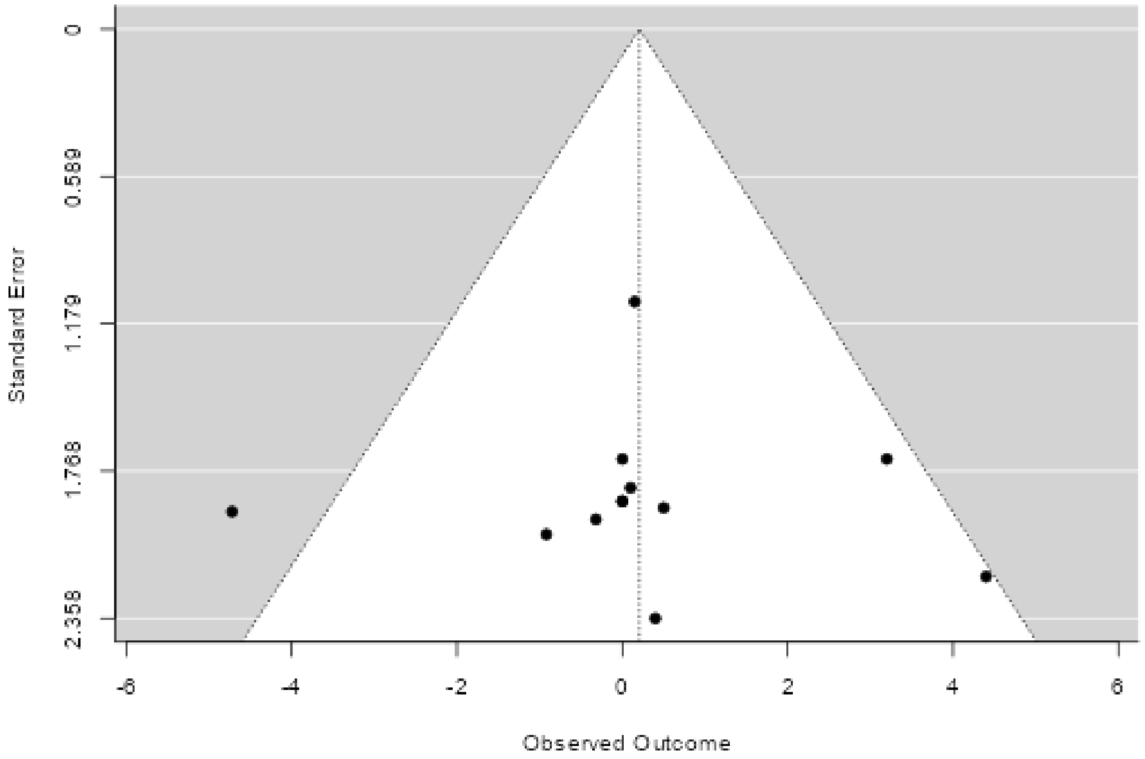

[3]

Two One-Sided Tests Equivalence Testing

Z-Value Lower Bound	P-Value Lower Bound	Z-Value Upper Bound	P-Value Upper Bound	LL_CI_TOST	UL_CI_TOST	LL_CI_ZTEST	UL_CI_ZTEST
1.379	0.084	-0.571	0.284	-0.636	1.050	-0.798	1.212

[4]

Equivalence Test Plot

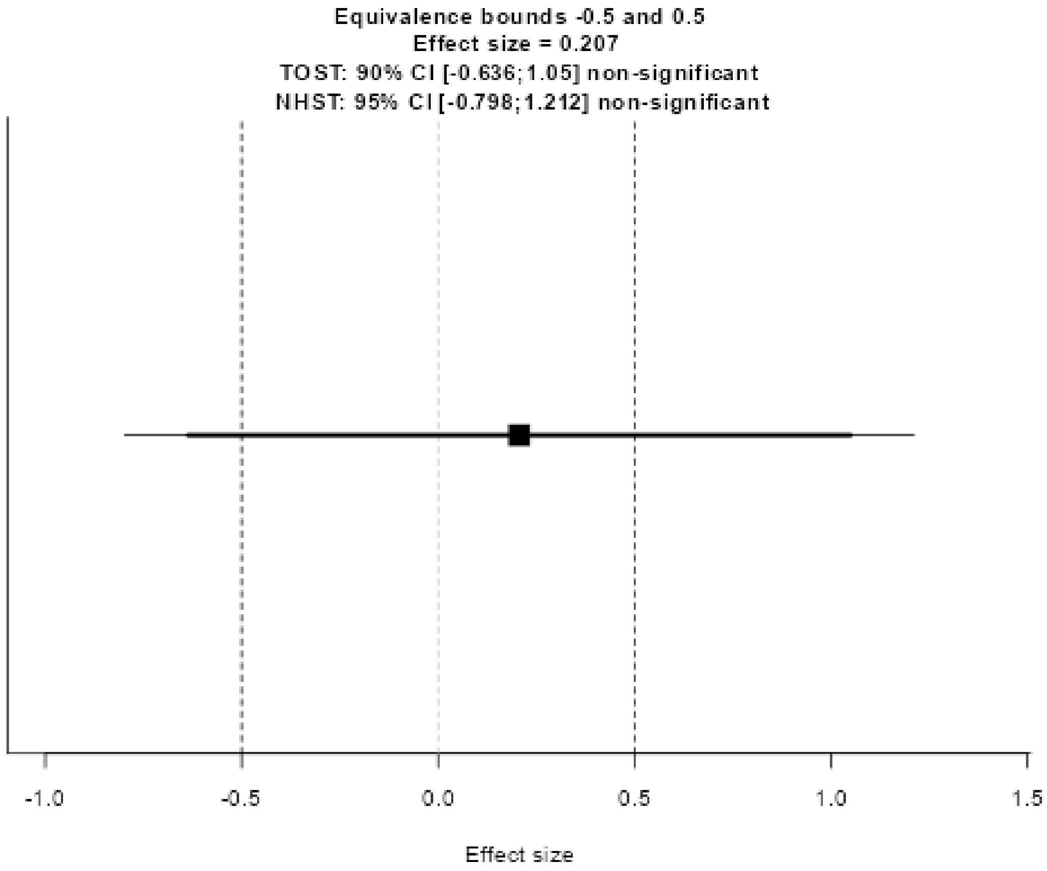

[4]

Referências

[1] The jamovi project (2022). *jamovi*. (Version 2.3) [Computer Software]. Retrieved from https://www.jamovi.org.

[2] R Core Team (2021). *R: A Language and environment for statistical computing*. (Version 4.1) [Computer software]. Retrieved from https://cran.r-project.org. (R packages retrieved from MRAN snapshot 2022-01-01).

[3] Viechtbauer, W. (2010). Conducting meta-analyses in R with the metafor package. *Journal of Statistical Software. link, 36*, 1-48.

[4] Lakens, D. (2017). Equivalence tests: A practical primer for t-tests, correlations, and meta-analyses. *Social Psychological and Personality Science. link, 1*, 1-8.
