# Supplementary material for: Validity and reliability of portable A-mode ultrasound in measuring body fat percentage: A systematic review with meta-analysis
Source: PLoS One. 2024 Feb 8;19(2):e0292872. doi: 10.1371/journal.pone.0292872 (PMC10852247; doi:10.1371/journal.pone.0292872)
Supplement: S4 File — (ZIP) [file pone.0292872.s005.zip › Statistical analysis supplement/Validity F random effect.pdf]

Results

Correlation Coefficients

Random-Effects Model (k = 5)

	Estimate	se	Z	p	CI Lower Bound	CI Upper Bound
Intercept	0.849	0.0291	29.2	< .001	0.792	0.906
	.	.	.	.	.	.

Nota. Tau^2 Estimator: Hunter-Schmidt

[3]

Heterogeneity Statistics

Tau	Tau^2	I^2	H^2	R^2	df	Q	p
0.038	0.0015 (SE= 0.0012)	35.41%	1.548	.	4.000	10.557	0.032

The analysis was carried out using the correlation coefficient as the outcome measure. A random-effects model was fitted to the data. The amount of heterogeneity (i.e., tau^2), was estimated using the Hunter-Schmidt estimator (Hunter 1990 ; Viechtbauer 2005). In addition to the estimate of tau^2, the Q-test for heterogeneity (Cochran 1954) and the I^2 statistic are reported. In case any amount of heterogeneity is detected (i.e., $tau^2 > 0$, regardless of the results of the Q-test), a prediction interval for the true outcomes is also provided. Studentized residuals and Cook's distances are used to examine whether studies may be outliers and/or influential in the context of the model. Studies with a studentized residual larger than the 100 x (1 - 0.05/(2 X k))th percentile of a standard normal distribution are considered potential outliers (i.e., using a Bonferroni correction with two-sided alpha = 0.05 for k studies included in the meta-analysis). Studies with a Cook's distance larger than the median plus six times the interquartile range of the Cook's distances are considered to be influential. The rank correlation test and the regression test, using the standard error of the observed outcomes as predictor, are used to check for funnel plot asymmetry.

A total of k=5 studies were included in the analysis. The observed correlation coefficients ranged from 0.6500 to 0.9010, with the majority of estimates being positive (100%). The estimated average correlation coefficient based on the random-effects model was \hat{\mu} = 0.8493 (95% CI: 0.7923 to 0.9064). Therefore, the average outcome differed significantly from zero (z = 29.1865, p < 0.0001). According to the Q-test, the true outcomes appear to be heterogeneous (Q(4) = 10.5574, p = 0.0320, tau^2 = 0.0015, I^2 = 35.4093%). A 95% prediction interval for the true outcomes is given by 0.7551 to 0.9436. Hence, even though there may be some heterogeneity, the true outcomes of the studies are generally in the same direction as the estimated average outcome. An examination of the studentized residuals revealed that one study (Johnson, K. E. et al., (2014) [49] [F]) had a value larger than ± 2.5758 and may be a potential outlier in the context of this model. According to the Cook's distances, none of the studies could be considered to be overly influential. The regression test indicated funnel plot asymmetry (p = 0.0056) but not the rank correlation test (p = 0.2333).

Forest Plot

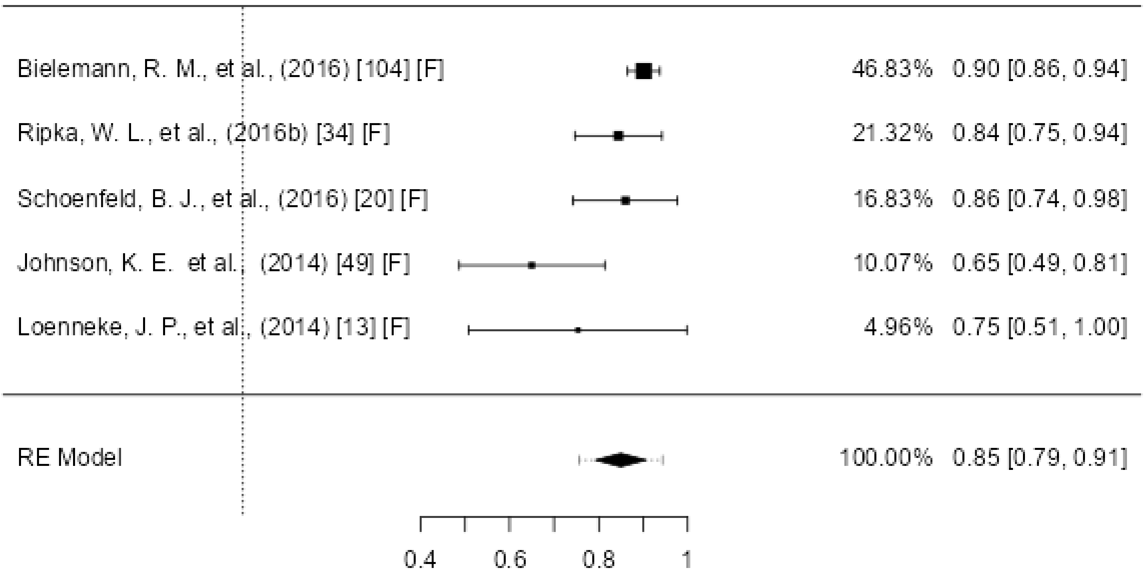

[3]

Publication Bias Assessment

Test Name	value	p
Fail-Safe N	3238.000	< .001
Begg and Mazumdar Rank Correlation	-0.600	0.233
Egger's Regression	-2.772	0.006
Trim and Fill Number of Studies	3.000	.

Nota. Fail-safe N Calculation Using the Rosenthal Approach

Funnel Plot

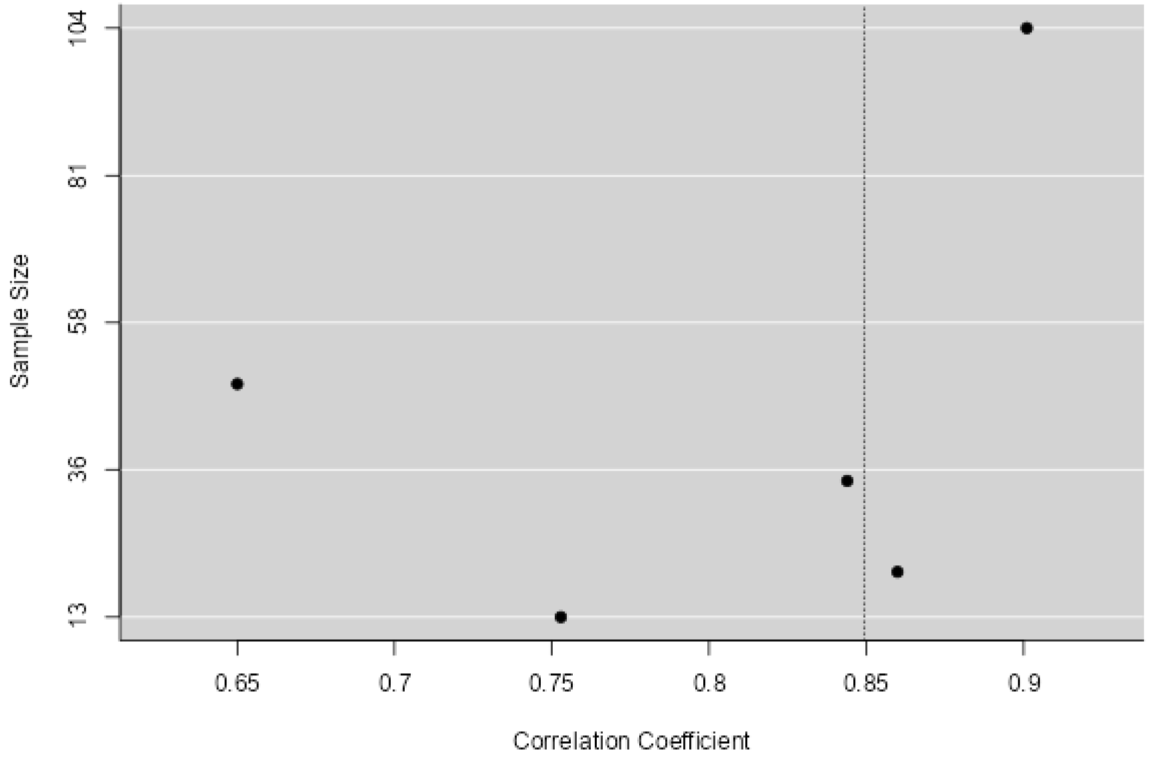

[3]

Referências

[1] The jamovi project (2022). *jamovi*. (Version 2.3) [Computer Software]. Retrieved from https://www.jamovi.org.

[2] R Core Team (2021). *R: A Language and environment for statistical computing*. (Version 4.1) [Computer software]. Retrieved from https://cran.r-project.org. (R packages retrieved from MRAN snapshot 2022-01-01).

[3] Viechtbauer, W. (2010). Conducting meta-analyses in R with the metafor package. *Journal of Statistical Software. link, 36*, 1-48.
