# Supplementary material for: Validity and reliability of portable A-mode ultrasound in measuring body fat percentage: A systematic review with meta-analysis
Source: PLoS One. 2024 Feb 8;19(2):e0292872. doi: 10.1371/journal.pone.0292872 (PMC10852247; doi:10.1371/journal.pone.0292872)
Supplement: S4 File — (ZIP) [file pone.0292872.s005.zip › Statistical analysis supplement/Validity r fixed effect.pdf]

Results

Correlation Coefficients

Fixed-Effects Model (k = 7)

	Estimate	se	Z	p	CI Lower Bound	CI Upper Bound
Intercept	0.818	0.0190	43.1	< .001	0.781	0.855
	.	.	.	.	.	.

[3]

Heterogeneity Statistics

Tau	Tau^2	I^2	H^2	R^2	df	Q	p
0.000	0 (SE= NA)	18.87%	1.233	.	6.000	7.396	0.286

The analysis was carried out using the correlation coefficient as the outcome measure. A fixed-effects model was fitted to the data. The Q-test for heterogeneity (Cochran 1954) and the I^2 statistic are reported. Studentized residuals and Cook's distances are used to examine whether studies may be outliers and/or influential in the context of the model. Studies with a studentized residual larger than the 100 x (1 - 0.05/(2 X k))th percentile of a standard normal distribution are considered potential outliers (i.e., using a Bonferroni correction with two-sided alpha = 0.05 for k studies included in the meta-analysis). Studies with a Cook's distance larger than the median plus six times the interquartile range of the Cook's distances are considered to be influential. The rank correlation test and the regression test, using the standard error of the observed outcomes as predictor, are used to check for funnel plot asymmetry.

A total of k=7 studies were included in the analysis. The observed correlation coefficients ranged from 0.6500 to 0.8790, with the majority of estimates being positive (100%). The estimated average correlation coefficient based on the fixed-effects model was \hat{\theta} = 0.8179 (95% CI: 0.7806 to 0.8551). Therefore, the average outcome differed significantly from zero (z = 43.0585, p < 0.0001). According to the Q-test, there was no significant amount of heterogeneity in the true outcomes (Q(6) = 7.3960, p = 0.2858, I^2 = 18.8749%). An examination of the studentized residuals revealed that none of the studies had a value larger than ± 2.6901 and hence there was no indication of outliers in the context of this model. According to the Cook's distances, none of the studies could be considered to be overly influential. Neither the rank correlation nor the regression test indicated any funnel plot asymmetry (p = 1.0000 and p = 0.3286, respectively).

Forest Plot

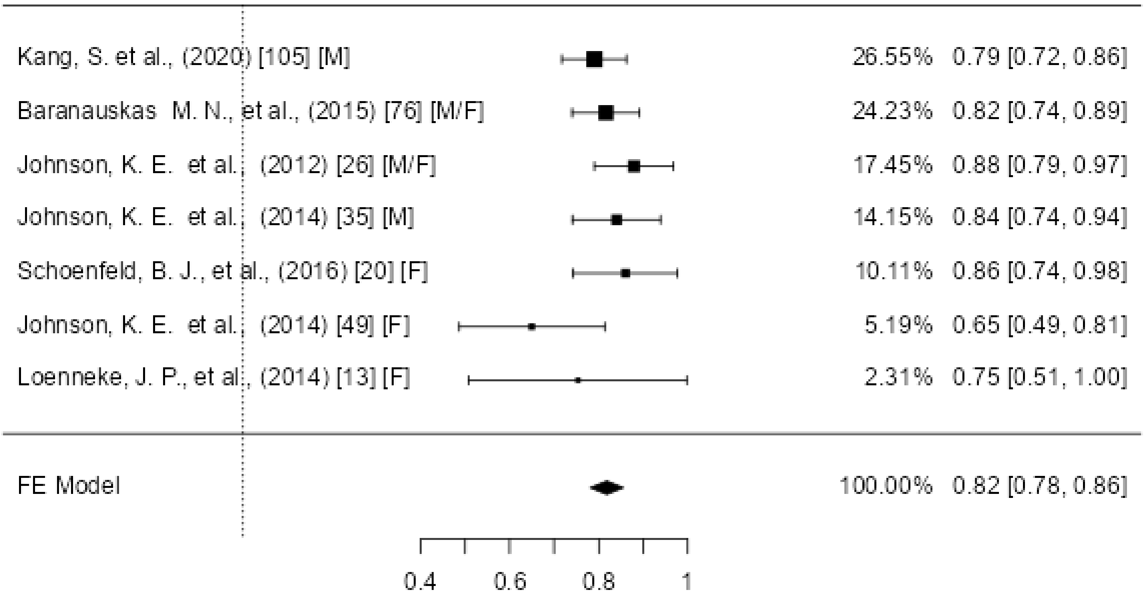

[3]

Publication Bias Assessment

Test Name	value	p
Fail-Safe N	4207.000	< .001
Begg and Mazumdar Rank Correlation	-0.048	1.000
Egger's Regression	-0.977	0.329
Trim and Fill Number of Studies	1.000	.

Nota. Fail-safe N Calculation Using the Rosenthal Approach

Funnel Plot

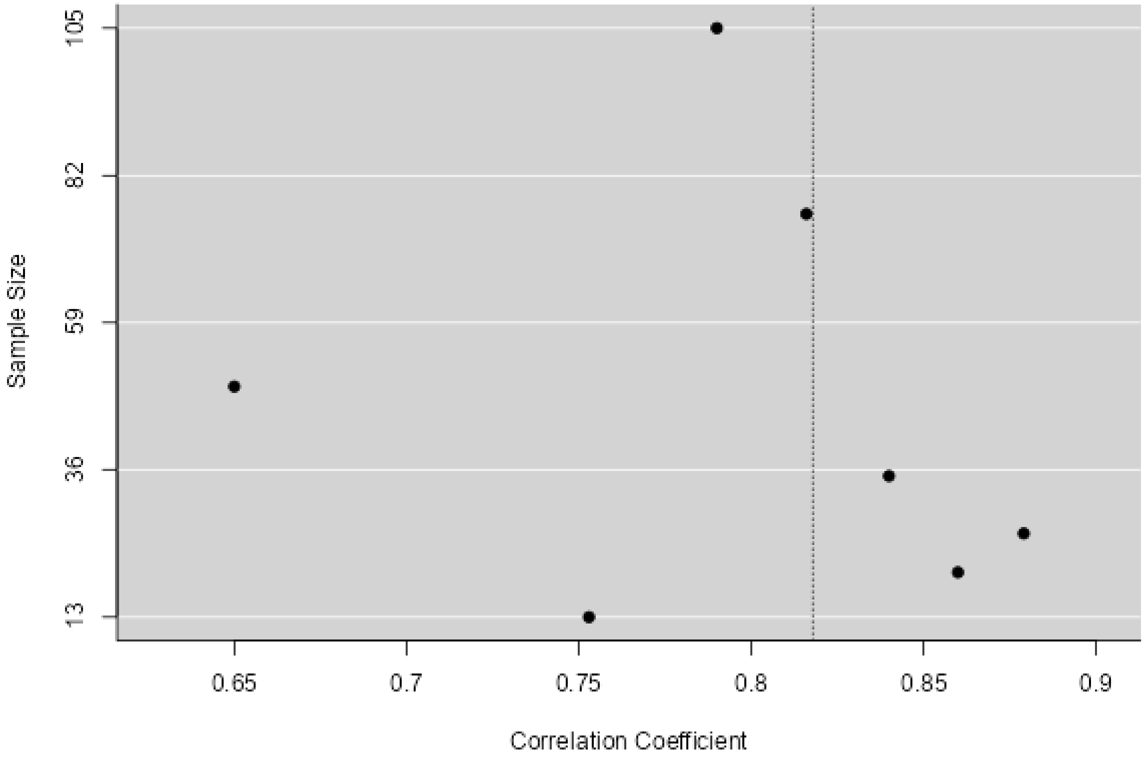

[3]

Referências

[1] The jamovi project (2022). *jamovi*. (Version 2.3) [Computer Software]. Retrieved from https://www.jamovi.org.

[2] R Core Team (2021). *R: A Language and environment for statistical computing*. (Version 4.1) [Computer software]. Retrieved from https://cran.r-project.org. (R packages retrieved from MRAN snapshot 2022-01-01).

[3] Viechtbauer, W. (2010). Conducting meta-analyses in R with the metafor package. *Journal of Statistical Software. link, 36*, 1-48.
