# Supplementary material for: Validity and reliability of portable A-mode ultrasound in measuring body fat percentage: A systematic review with meta-analysis
Source: PLoS One. 2024 Feb 8;19(2):e0292872. doi: 10.1371/journal.pone.0292872 (PMC10852247; doi:10.1371/journal.pone.0292872)
Supplement: Attachment — Submitted filename: Peer review.docx [file pone.0292872.s006.docx]

The authors made some progress in the meta-analysis and systematic review of Bodymetrix® branded portable ultrasound (P-US) measurement of body fat percentage, supplementing our previous understanding of how to use P-US to measure body fat percentage and suggesting that we have important implications for better, faster and more accurate measurement of body fat percentage. However, there are still some issues that need further improvement, as described below.

Major Issues

1. Lines 105-108, in the data standard, what is the specific method of P-UB compared with DXA, ADP, HW methods? Are the resulting BF% values true and comparable? On what basis?

2. Lines 44-47, read whether the observer used BodyMetrix™ to compare Three-Site and Seven-Site measurements, suggesting further refinement of the Seven-Site statistical analysis data.

3. Line 154, The QUADAS-2 in the design method is proposed to be modified to QUADAS-C (Quality Assessment of Diagnostic Accuracy Studies-Comparative). The QUADAS-C tool was developed as an extension of QUADAS-2 to assess the risk of bias in comparative diagnostic test accuracy studies.

Minor Issues

4. Lines 100-101, 123-125, 218, refer to the website address as deleted or abbreviated as possible

5. In the flow diagram in Figure 1, the changes are corrected in the order of Identification、Screening、Eligibility、Included.

6. In Figuer3 and Figuer5, it is recommended to modify the setting position of Result in the figure, and do not overlap with other contents

Peer reviewer：Cui hai
