# Supplementary material for: Validity and reliability of portable A-mode ultrasound in measuring body fat percentage: A systematic review with meta-analysis
Source: PLoS One. 2024 Feb 8;19(2):e0292872. doi: 10.1371/journal.pone.0292872 (PMC10852247; doi:10.1371/journal.pone.0292872)
Supplement: Attachment — Submitted filename: Response to Reviewers.docx [file pone.0292872.s007.docx]

In order to respond to the editor’s and reviewers’ suggestions, the changes and arguments regarding the comments are presented below:

**Comment from the Academic Editor:**

Change in the purpose of the study, “Evaluate the validity and reliability of the P-US for measuring body fat percentage (BF%)”.

Response to Academic Publisher’s: In view of the comment, we decided to change the study objective, as suggested.

**Reviewers' comments.**

1. Is the manuscript technically sound, and do the data support the conclusions?

Reviewer #1: Yes

Reviewer #2: Yes

Response to Reviewers: no comment.

2. Has the statistical analysis been performed appropriately and rigorously?

Reviewer #1: Yes

Reviewer #2: Yes

Response to Reviewers: no comments, thanks!

3. Have the authors made all data underlying the findings in their manuscript fully available?

Reviewer #1: No

Reviewer #2: Yes

Response to Reviewers: In response to Reviewer 1's comment, we decided to insert the data sheets provided by the JAMOVI software for the validity and reliability results, the analysis of the QUADAS-2 instrument, as well as that of the QAREL (Supplement 2).

4. Is the manuscript presented in an intelligible fashion and written in standard English?

Reviewer #1: No, review, please.

Reviewer #2: Yes

Response to Reviewers: In response to Reviewer 1's comment, we decided to forward the manuscript to a professional native English (Canadian) reviewer for further review.

5. Review Comments to the Author

Reviewer #1: The authors made some progress in the meta-analysis and systematic review of Bodymetrix® branded portable ultrasound (P-US) measurement of body fat percentage, supplementing our previous understanding of how to use P-US to measure body fat percentage and suggesting that we have important implications for better, faster and more accurate measurement of body fat percentage.

Reviewer #2: This paper addresses an interesting problem- whether Portable Ultrasound (P-US) is a valid and reliable method to measure body fat percentage (BF%). The authors propose systematic review with Meta-analysis study to review and synthesize the existing literatures. The results show high validity and reliability of the P-US as a device for measuring body fat percentage. Publication bias and subgroups analysis are also performed. Overall, the article is well organized and the statistical analysis is rigorous and sound.

Response to Reviewers: no comments, thanks!

6. PLOS authors have the option to publish the peer review history of their article (what does this mean?). If published, this will include your full peer review and any attached files

If you choose “no”, your identity will remain anonymous but your review may still be made public.

Do you want your identity to be public for this peer review? For information about this choice, including consent withdrawal, please see our Privacy Policy.

Reviewer #1: Yes

Reviewer #2: No

In response to questions raised by Reviewer 1 through the file “Peer review.docx”.

The authors made some progress in the meta-analysis and systematic review of Bodymetrix® branded portable ultrasound (P-US) measurement of body fat percentage, supplementing our previous understanding of how to use P-US to measure body fat percentage and suggesting that we have important implications for better, faster and more accurate measurement of body fat percentage. However, there are still some issues that need further improvement, as described below.

**Major Issues**

1. Lines 105-108, in the data standard, what is the specific method of P-UB compared with DXA, ADP, HW methods? Are the resulting BF% values true and comparable? On what basis?

Lines 105-108: “Considering the PIRO^(21)^ strategy, studies were selected with participants of both genders, of any age (Participant), who assessed body composition with the P-US of the Bodymetrix^®^ brand (Index Test) in comparison with the DXA, or the ADP or even the HW (Reference Standard) and that presented results for the BF% (Outcome)”.

Reply to Reviewer 1:

In response to the question raised, we decided to characterize the way of decoding the signals emitted by the ultrasound, inserting the term “A-mode” (amplitude), characteristic of the Portable Ultrasound A-mode (US-P) of the brand Bodymetrix^®^ (IntelaMetrix, Inc – model: BX 2000). We inserted the term “A-mode” in lines 4, 23, 75, and 95.

Although DXA, ADP, and HW are established as valid methods for assessing body composition, and in particular fat percentage, we recognize that there is still no consensus in the literature regarding the reliability between the DXA and ADP methods.

Thus, in lines 111-113, we inserted following wording: “In the present review, we consider the results of DXA and ADP as valid and reliable for determining body composition in the presented outcome (inserting 4 references)”.

Note: in the Discussion item, in the reliability studies, it was verified that the meta-analysis was able to mitigate the possible lack of agreement between the DXA and ADP instruments. We have inserted the following wording (lines 385-388): “Even admitting the possibility of inaccuracy between the DXA and PDA instruments, admitted as the reference standard, it seems that the meta-analysis was able to mitigate the incompatibility between them”.

2. Lines 44-47, read whether the observer used BodyMetrix™ to compare Three-Site and Seven-Site measurements, suggesting further refinement of the Seven-Site statistical analysis data.

Lines 44-47: “However, the P-US measures satisfactorily when it measures up to three sites associated with anthropometric perimeters in Multiple Linear Regression Equations with samples greater than 100 participants, and males”.

Reply to Reviewer 1:

The reports in lines 44-47 reinforce that the best results presented by the meta-analysis are those that used prediction equations involving the P-US measuring up to three sites associated with anthropometric measurements with samples greater than 100 male participants.

It is possible that we didn’t word it properly, so we apologize. Thus, in consideration of the Reviewer, we decided to change the wording: “The P-US associated with anthropometric perimeters satisfactorily measures the BF% with samples greater than 100 participants, and males”.

3. Line 154, The QUADAS-2 in the design method is proposed to be modified to QUADAS-C (Quality Assessment of Diagnostic Accuracy Studies-Comparative). The QUADAS-C tool was developed as an extension of QUADAS-2 to assess the risk of bias in comparative diagnostic test accuracy studies.

Line 154-158: QUADAS-2^(24)^ was used to assess the risk of bias in the validity studies, which is structured so that the key domains (Patient Selection; Index Test(s); Reference Standard; Flow and Time) are classified in terms of risk of bias and research applicability concerns. Each key domain presents a series of guiding questions that help in judging biases and applicability, as shown in Figure 2.

Reply to Reviewer 1:

To respond to the Reviewer's request, we turned to Yang et al. (2020) and some published reviews (YOON et al., 2023; CHOW et al., 2023; TSIKOPOULOS et al., 2022; ZYFODIA et al., 2021) to support our position.

The QUADAS-C tool (Quality Assessment of Diagnostic Accuracy Studies-Comparative) was developed as an extension of QUADAS-2 to assess the risk of bias in comparative studies of diagnostic accuracy when evaluating the accuracy of 2 or more Index Tests compared to each other and to the Reference Standard. The “C” stands for comparison when assessing the risk of bias in studies included in a systematic review. Table 1, below, presents the characteristics of the aforementioned studies compared with our manuscript.

Table 1: Comparison between different publications

| Author (year) | Index Tests  (ITa vs ITb) | Reference Standard |
| --- | --- | --- |
| Yoon et al. (2023) | ITa: AI  ITb: Evaluation by Radiologists | Tomography |
| Chow et al. (2023) | iTa: PSMA-PET  ITb: CIM | Histopathology |
| Tsikopoulos et al. (2022) | iTa: DTT  ITb: Sonification | Musculoskeletal Infection Society Criteria |
| Zyfodia et al. (2021) | iTa: Xpert Ultra  ITb: Xpert MTB | Rifacipin resistance |
| Our manuscript | iTa: P-US  ITb: | DXA or ADP or HW |

ITa: Index Test a; ITb: Index Test b; AI: Artificial intelligence; PSMA-PET: positron emission tomography of prostate-specific membrane antigen; CIM: Conventional Imaging Modalities; DTT: dithiothreitol; Xpert Ultra: rapid test to detect tuberculosis; Xpert MTB: rapid test to detect tuberculosis; P-US: Portable Ultrasound; DXA: Dual-energy X-ray Absorptiometry; ADP: Air Displacement Plethysmography; HW: Hydrostatic Weighing.

Our manuscript uses only one IT (P-US), there are no comparisons with other index tests, we evaluate its results with the Reference Standard (DXA or ADP, or HW). By associating anthropometric perimeters with the P-US results in prediction equations, we do not consider this procedure as another instrument for evaluating BF%. We understand that QUADAS-C would be necessary if studies were located that evaluated the performance between P-US (ITa) and bioimpedance (ITb), having DXA as the Reference Standard, a hypothetical study that has not been located so far.

These being the arguments and with the necessary respect, we disagree with Reviewer 1's suggestion.

**Minor Issues**

4. Lines 100-101, 123-125, 218, refer to the website address as deleted or abbreviated as possible

Reply to Reviewer 1:

Because of the suggestions, we decided to remove the electronic addresses.

100-101 [www.prisma-statment.org](http://www.prisma-statment.org)

123 [www.decs.bvsalud.org/en/](http://www.decs.bvsalud.org/en/)

125 <https://www.ncbi.nlm.nih.gov/mesh/>

5. In the flow diagram in Figure 1, the changes are corrected in the order of Identification、Screening、Eligibility、Included.

Reply to Reviewer 1:

Because of the suggestions, we have corrected the flowchart.

6. In Figure 3 and Figure 5, it is recommended to modify the setting position of Result in the figure, and do not overlap with other contents

Reply to Reviewer 1:

Taking into account the suggestions, we have improved the image.

REFERENCES

Yang B, Mallett S, Takwoingi Y, Davenport CF, Hyde CJ, Whiting PF, Deeks JJ, Leeflang MMG; QUADAS-C Group†; Bossuyt PMM, Brazzelli MG, Dinnes J, Gurusamy KS, Jones HE, Lange S, Langendam MW, Macaskill P, McInnes MDF, Reitsma JB, Rutjes AWS, Sinclair A, de Vet HCW, Virgili G, Wade R, Westwood ME. QUADAS-C: A Tool for Assessing Risk of Bias in Comparative Diagnostic Accuracy Studies. **Ann Intern Med.** 2021 Nov;174(11):1592-1599. doi: 10.7326/M21-2234. Epub 2021 Oct 26. PMID: 34698503.

Yoon JH, Strand F, Baltzer PAT, Conant EF, Gilbert FJ, Lehman CD, Morris EA, Mullen LA, Nishikawa RM, Sharma N, Vejborg I, Moy L, Mann RM. Standalone AI for Breast Cancer Detection at Screening Digital Mammography and Digital Breast Tomosynthesis: A Systematic Review and Meta-Analysis. **Radiology**. 2023 Jun;307(5):e222639. doi: 10.1148/radiol.222639. Epub 2023 May 23. PMID: 37219445; PMCID: PMC10315526.

Chow KM, So WZ, Lee HJ, Lee A, Yap DWT, Takwoingi Y, Tay KJ, Tuan J, Thang SP, Lam W, Yuen J, Lawrentschuk N, Hofman MS, Murphy DG, Chen K. Head-to-head Comparison of the Diagnostic Accuracy of Prostate-specific Membrane Antigen Positron Emission Tomography and Conventional Imaging Modalities for Initial Staging of Intermediate- to High-risk Prostate Cancer: A Systematic Review and Meta-analysis. **Eur Urol**. 2023 Jul;84(1):36-48. doi: 10.1016/j.eururo.2023.03.001. Epub 2023 Apr 7. PMID: 37032189.

Tsikopoulos K, Christofilos SI, Kitridis D, Sidiropoulos K, Stoikos PN, Gravalidis C, Givissis P, Papaioannidou P. Is sonication superior to dithiothreitol in diagnosis of periprosthetic joint infections? A meta-analysis. Int Orthop. 2022 Jun;46(6):1215-1224. doi: 10.1007/s00264-022-05350-z. Epub 2022 Feb 24. Erratum in: **Int Orthop**. 2022 Mar 24;: PMID: 35199219.

Zifodya JS, Kreniske JS, Schiller I, Kohli M, Dendukuri N, Schumacher SG, Ochodo EA, Haraka F, Zwerling AA, Pai M, Steingart KR, Horne DJ. Xpert Ultra versus Xpert MTB/RIF for pulmonary tuberculosis and rifampicin resistance in adults with presumptive pulmonary tuberculosis. **Cochrane Database Syst Rev**. 2021 Feb 22;2:CD009593. doi: 10.1002/14651858.CD009593.pub5. PMID: 33616229.
