# Supplementary material for: Validity and reliability of portable A-mode ultrasound in measuring body fat percentage: A systematic review with meta-analysis
Source: PLoS One. 2024 Feb 8;19(2):e0292872. doi: 10.1371/journal.pone.0292872 (PMC10852247; doi:10.1371/journal.pone.0292872)
Supplement: Attachment — Submitted filename: Peer review.docx [file pone.0292872.s008.docx]

The authors made some progress in the meta-analysis and systematic review of Bodymetrix® branded portable ultrasound (P-US) measurement of body fat percentage, supplementing our previous understanding of how to use P-US to measure body fat percentage and suggesting that we have important implications for better, faster and more accurate measurement of body fat percentage. However, there are still some issues that need further improvement, as described below.

Minor Issue

1. Lines 214-215, It is recommended to delete the connection to the JAMOVI 2.2.5 software program download site, as any update or change may lead to misunderstanding by readers or researchers.

2. Figure2, Modify the color distinction in Figure2 to be consistent with that in Table 1. Different risk levels are suggested to be expressed in numbers (e.g: low risk 1, high risk 2, unknown risk 3).

3. Table 3, Change the data format of the Heterogeneity and Meta-analysis items in Table 3 to left-justified

Peer reviewer：Cui hai
