# Supplementary material for: Validity and reliability of portable A-mode ultrasound in measuring body fat percentage: A systematic review with meta-analysis
Source: PLoS One. 2024 Feb 8;19(2):e0292872. doi: 10.1371/journal.pone.0292872 (PMC10852247; doi:10.1371/journal.pone.0292872)
Supplement: Attachment — Submitted filename: Response to Reviewer.docx [file pone.0292872.s009.docx]

In order to respond to the reviewer’s suggestions, the changes and arguments regarding the comments are presented below:

**Comment from Reviewer #1:**

1. If the authors have adequately addressed your comments raised in a previous round of review and you feel that this manuscript is now acceptable for publication, you may indicate that here to bypass the “Comments to the Author” section, enter your conflict of interest statement in the “Confidential to Editor” section, and submit your "Accept" recommendation.

Reviewer #1: All comments have been addressed.

**Reply to Reviewer #1:** thanks!

2. Is the manuscript technically sound, and do the data support the conclusions?

Reviewer #1: Yes

**Reply to Reviewer #1:** no comment

3. Has the statistical analysis been performed appropriately and rigorously?

Reviewer #1: I Don't Know

**Reply to Reviewer #1:** We inform you that the statistical analysis used was appropriate for the following reasons:

3.1. The JAMOVI program has already been used in other studies^(1–5)^ and is capable of analyzing mean effect measures and correlations;

3.2. The selection of effect measures was appropriate: for validity, Pearson’s correlation coefficient; for reliability, the result of the Bland-Altman Test, which is the mean of the errors;

3.3. Heterogeneity was assessed for both outcomes, and according to its results, the analysis model (fixed effect or random effects) was used to incorporate it or not into the meta-analysis and explain it;

3.4. In the case of high and true heterogeneity, subgroup analyzes were performed in an attempt to explain it;

3.5. All of this information is clearly described in the Effect measures and Synthesis of collected data methods (lines 184-246) in the Method section.

4. Have the authors made all data underlying the findings in their manuscript fully available?

Reviewer #1: Yes

**Reply to Reviewer #1:** no comment.

5. Is the manuscript presented in an intelligible fashion and written in standard English?

Reviewer #1: Yes

**Reply to Reviewer #1:** no comment, thanks!

6. Review Comments to the Author

Reviewer #1: The authors made some progress in the meta-analysis and systematic review of Bodymetrix^®^ branded portable ultrasound (P-US) measurement of body fat percentage, supplementing our previous understanding of how to use P-US to measure body fat percentage and suggesting that we have important implications for better, faster and more accurate measurement of body fat percentage.

**Reply to Reviewer #1:** no comment.

7. PLOS authors have the option to publish the peer review history of their article ([what does this mean?](https://journals.plos.org/plosone/s/editorial-and-peer-review-process#loc-peer-review-history)). If published, this will include your full peer review and any attached files.

If you choose “no”, your identity will remain anonymous but your review may still be made public.

**Do you want your identity to be public for this peer review?** For information about this choice, including consent withdrawal, please see our [Privacy Policy](https://www.plos.org/privacy-policy).

Reviewer #1: Yes: Hai Cui

The authors made some progress in the meta-analysis and systematic review of Bodymetrix^®^ branded portable ultrasound (P-US) measurement of body fat percentage, supplementing our previous understanding of how to use P-US to measure body fat percentage and suggesting that we have important implications for better, faster and more accurate measurement of body fat percentage. However, there are still some issues that need further improvement, as described below.

**Minor Issue**

1. Lines 214-215, It is recommended to delete the connection to the JAMOVI 2.2.5 software program download site, as any update or change may lead to misunderstanding by readers or researchers.

**Reply to Reviewer #1:** in response to the suggestion, we decided to remove the JAMOVI software version and web address.

2. Figure 2, Modify the color distinction in Figure 2 to be consistent with that in Table 1. Different risk levels are suggested to be expressed in numbers (e.g: low risk 1, high risk 2, unknown risk 3).

**Reply to Reviewer #1:** in response to the suggestion, we standardized the colors based on Table 1 and inserted the numbers 1, 2 and 3 for “low risk”, “high risk” and “uncertain risk”, respectively.

3. Table 3, Change the data format of the Heterogeneity and Meta-analysis items in Table 3 to left-justified.

**Reply to Reviewer #1:** We changed the formatting of the table, as suggested.

References:

1.	Leporace G, Metsavaht L, Gonzalez FF, Arcanjo de Jesus F, Machado M, Celina Guadagnin E, et al. Validity and reliability of two-dimensional video-based assessment to measure joint angles during running: A systematic review and meta-analysis. J Biomech [Internet]. 2023;157(July):111747. Available at: https://doi.org/10.1016/j.jbiomech.2023.111747.

2.	Dokponou YCH, Badirou OBA, Agada KN, Dossou MW, Lawson LD, Ossaga MAD, et al. Transcranial doppler in the non-invasive estimation of intracranial pressure in traumatic brain injury compared to other non-invasive methods in lower-middle income countries: Systematic review and meta-analysis. J Clin Neurosci. 2023;113(May):70–6.

3.	Pentapati KC, Yeturu SK, Siddiq H. A reliability generalization meta-analysis of Child Oral Impacts on Daily Performances (C – OIDP) questionnaire. J Oral Biol Craniofacial Res [Internet]. 2020;10(4):776–81. Available at: https://doi.org/10.1016/j.jobcr.2020.10.017

4.	Farzan R, Hosseini SJ, Firooz M, Tabarian MS, Jamshidbeigi A, Samidoust P, et al. Perceived stigmatisation and reliability of questionnaire in the survivors with burns wound: A systematic review and meta-analysis. Int Wound J. 2023;(March):1–13.

5.	Olumade TJ, Uzairue LI. Clinical characteristics of 4499 COVID-19 patients in Africa: A meta-analysis. J Med Virol. 2021;93(5):3055–61.
